# Customized bioreactor enables the production of 3D diaphragmatic constructs influencing matrix remodeling and fibroblast overgrowth

**DOI:** 10.1038/s41536-022-00222-x

**Published:** 2022-04-25

**Authors:** Edoardo Maghin, Eugenia Carraro, Daniele Boso, Arben Dedja, Mattia Giagante, Paola Caccin, Raluca Ana-Maria Barna, Silvia Bresolin, Alice Cani, Giulia Borile, Deborah Sandrin, Filippo Romanato, Francesca Cecchinato, Anna Urciuolo, Dorianna Sandonà, Paolo De Coppi, Piero G. Pavan, Martina Piccoli

**Affiliations:** 1 Tissue Engineering Lab, Fondazione Istituto di Ricerca Pediatrica Città della Speranza, Padova, Italy; 2 Department of Biomedical Sciences, University of Padova, Padova, Italy; 3 Department of Industrial Engineering, University of Padova, Padova, Italy; 4 Department of Cardiac, Thoracic and Vascular Sciences and Public Health, University of Padova, Padova, Italy; 5 Onco-Hematology Lab, Department of Women’s and Children’s Health, University of Padova, Padova, Italy; 6 Optics and Bioimaging Lab, Fondazione Istituto di Ricerca Pediatrica Città della Speranza, Padova, Italy; 7 Department of Physics and Astronomy, University of Padova, Padova, Italy; 8 L.I.F.E.L.A.B. Program, Consorzio per la Ricerca Sanitaria (CORIS), Veneto Region, Padova, Italy; 9 Neuromuscular Engineering Lab, Fondazione Istituto di Ricerca Pediatrica Città della Speranza, Padova, Italy; 10 Molecular Medicine Department, University of Padova, Padova, Italy; 11 Department of Specialist Neonatal and Pediatric Surgery, Great Ormond Street Hospital, London, UK; 12 Stem Cells and Regenerative Medicine Section, Department of Pediatric Surgery, UCL Great Ormond Street Institute of Child Health, London, UK

**Keywords:** Tissue engineering, Regenerative medicine, Biomaterials - cells

## Abstract

The production of skeletal muscle constructs useful for replacing large defects in vivo, such as in congenital diaphragmatic hernia (CDH), is still considered a challenge. The standard application of prosthetic material presents major limitations, such as hernia recurrences in a remarkable number of CDH patients. With this work, we developed a tissue engineering approach based on decellularized diaphragmatic muscle and human cells for the in vitro generation of diaphragmatic-like tissues as a proof-of-concept of a new option for the surgical treatment of large diaphragm defects. A customized bioreactor for diaphragmatic muscle was designed to control mechanical stimulation and promote radial stretching during the construct engineering. In vitro tests demonstrated that both ECM remodeling and fibroblast overgrowth were positively influenced by the bioreactor culture. Mechanically stimulated constructs also increased tissue maturation, with the formation of new oriented and aligned muscle fibers. Moreover, after in vivo orthotopic implantation in a surgical CDH mouse model, mechanically stimulated muscles maintained the presence of human cells within myofibers and hernia recurrence did not occur, suggesting the value of this approach for treating diaphragm defects.

## Introduction

Skeletal muscle is a finely organized tissue, in which myoblasts form highly aligned muscle fibers through fusion into multi-nucleated myotubes^1^. This peculiar arrangement, together with tissue-specific extracellular matrix (ECM), is essential for the generation of contractile force^2^. For some years, in vitro 3D muscle constructs have been developed to investigate suitable biologic substitutes for in vivo muscle replacement^3^, or to study mechanisms behind aging and degeneration rather than to understand the tissue response following the administration of therapeutic molecules^4,5^. In vitro engineered skeletal muscle tissues generally consist of synthetic or biologic scaffolds embedded with stem or progenitor cells. Multiple approaches have been proposed to generate 3D skeletal muscles, from the use of myogenic progenitors to induced pluripotent stem (iPS)-derived cells as a cellular component^6–10^, and from polyethylene glycol (PEG)-derived supports to fibrin hydrogels as scaffold^11–14^. The choice of scaffold origin and composition is crucial, since physical aspects of the microenvironment are strongly linked to gene expression and protein organization of the embedded cells^15,16^. ECM derived scaffolds have not only the advantage of offering mechanical support in keeping with skeletal muscle characteristics, but also play a major role in conditioning cellular behavior through retaining the chemical compositions of the original tissue^17,18^. For this reason, many studies have focused on in vitro muscle regeneration using different ECM scaffold formulations obtained through tissue decellularization^19–21^. However, there are still limitations related to biological variability and cell seeding, and there is a need to develop technology to help standardized recellularization processes and physiological regenerations.

Given the pivotal role of environmental factors in conditioning cell behavior, some of the experimental protocols, together with cells and scaffolds, include the use of devices to apply mechanical (tensile strain) or electrical stimulation as microenvironmental enrichment for muscle cell alignment during myotube formation^13,22,23^. Static strain and specific scaffold compliance partially recapitulate the embryonic muscle environment mimicking the effect of bone elongation during development^24,25^, and have been demonstrated to increase cell alignment, fusion and expression of myogenic genes^12,26^. On the other hand, cyclic strain studies have reported discordant results, probably depending on the timing at which the mechanical stimulus was applied^27,28^. To date, these in vitro approaches have principally been performed with the final aim of producing a linear muscle, following a single axis of strain and orientation that in several cases leads to a single axis of contraction^13,29^. However, this classic unidirectional mechanical stimulus is no longer efficient when the goal is to generate in vitro a muscle with differently organized or mixed, but ordered, myofiber orientations such as in the diaphragm.

Indeed, unlike other human skeletal muscles, the diaphragm, an essential muscle with a critical function in supporting respiration, presents the unique anatomy of a flat shape, with a central tendon and outwardly radiating myofibers. A wide range of injuries affecting the diaphragmatic muscle are encountered clinically: from genetic (the diaphragm is the most affected muscle in muscular dystrophies^30^) to congenital (i.e. diaphragmatic hernia^31^) or mechanical (due to accidents or surgical resections of a tumor such as pleural mesotheliomas^32^). In this context, the treatment of congenital diaphragmatic hernia (CDH) is particularly demanding as it affects 1 per 2500 live births. In newborns, when the missing diaphragm cannot be closed with primary sutures, patch implantation is needed. Nowadays, polytetrafluorethylene (PTFE) and Goretex^®^ are the most widely used materials for the closure of large CDH defects^33^. Unfortunately, in the case of a large defect or agenesis of the diaphragm, the application of prosthetic material has strong limitations, such as hernia recurrences and the need for multiple surgeries to replace the diaphragm patch^34,35^. Besides recurrence, the role of non-absorbable implants in long-term recognized complications after CDH repairs such as scoliosis and bowel obstruction is unclear^36^. There are two mandatory requirements for optimal tissue-engineered substitutes that aim at overcoming the drawbacks of the current approaches^37^: suitable mechanical tensile strength to withstand muscle deformation due to the child’s growth, and the ability to integrate with the host diaphragm for the rest of the patient’s life.

Recently, we generated in vitro a viable and functional construct obtained by the combination of mouse decellularized diaphragm ECM (dECM) and human muscle progenitor cells^38^. In that work, the diaphragm-like tissue demonstrated not only the maintenance of a heterogeneous pool of myogenic cells (from PAX7-positive stem cells to fully differentiated and metabolic active myofibers), but also a precise response to external stimuli, such as myotoxin treatment. Predictably, in that model, the newly generated myofibers randomly aligned, due to the low specific directional stimulation inside the tissue culture.

To overcome this issue, in this research work, we designed and fabricated a diaphragm-specific bioreactor to radially stimulate diaphragmatic constructs with programmable and individually tunable control of strain parameters. The final goal was to achieve wider spread of the cells and to increase their alignment inside a dECM scaffold by applying a slow radial tensile strain for several days, and to improve construct maturity and functionality through the application of cyclic mechanical strain as a training period. We performed static and dynamic experiments in parallel and evaluated the differences in terms of cell distribution, differentiation, and maturation at molecular and protein levels, demonstrating the importance of specific mechanical stimulation to obtain physiologically aligned and mature 3D diaphragmatic constructs with better performance. Finally, we orthotopically implanted the 3D dynamic constructs in vivo to mimic a possible treatment of CDH.

## Results

### 3D diaphragmatic engineering using human skeletal muscle cells, human fibroblasts, and decellularized mouse diaphragm ECM

**Fig. 1:**
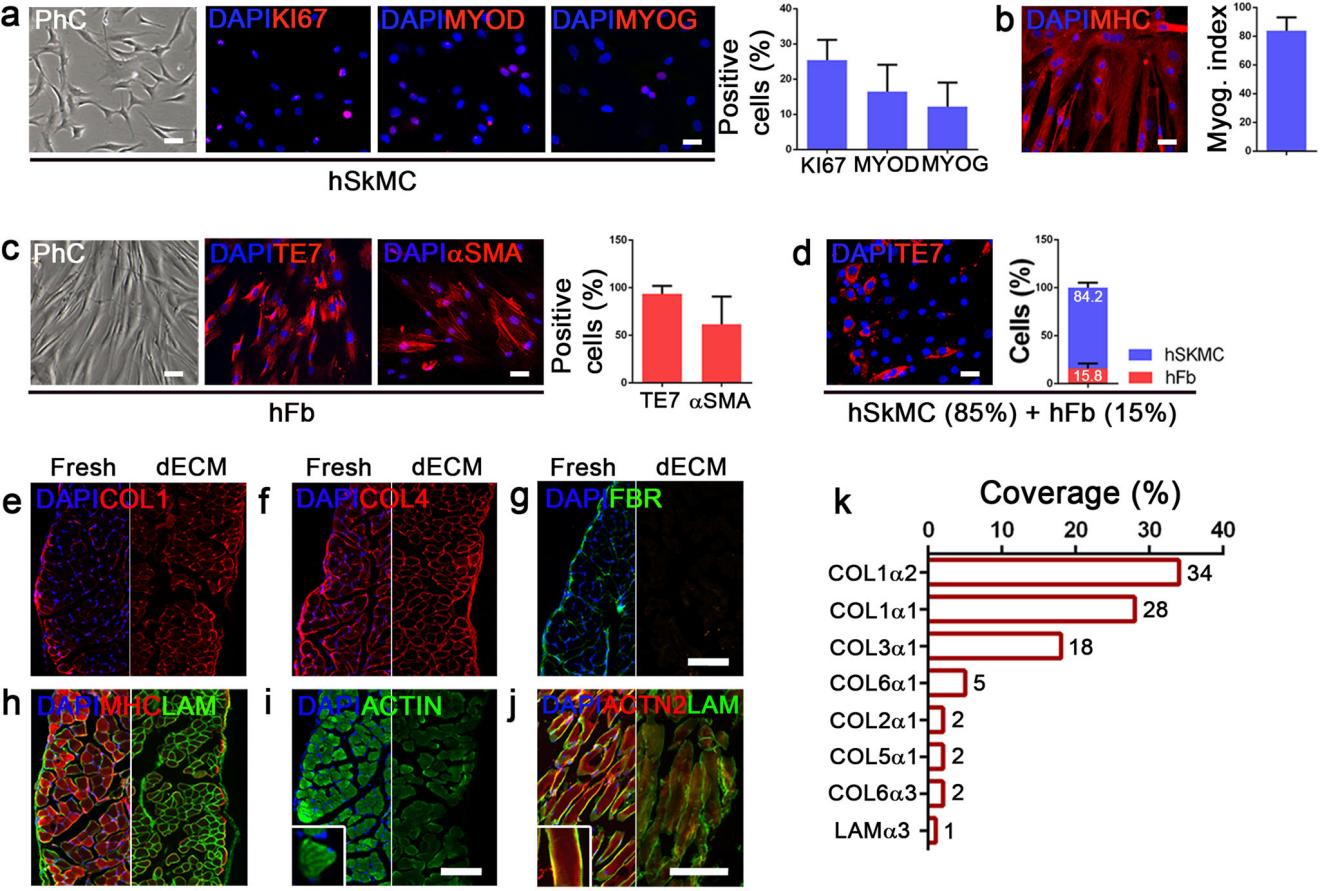
Diaphragmatic-like construct components. **a** Human skeletal muscle cells (hSkMC) characterization: immunofluorescence and quantification of cells positive for a proliferation marker (KI67) and myogenic markers (MYOD and MYOG; *n* = 4, mean ± s.d.). **b** Immunofluorescence of MHC and quantification of myogenic index (*n* = 4; mean ± s.d.). **c** Human fibroblasts characterization (hFb): immunofluorescence and quantification of TE7 and αSMA positive cells (*n* = 4). **d** Immunofluorescence and quantification of TE7-positive cells in 2D co-cultures of hSkMC (85%) and hFb (15%) after 4 days (*n* = 4; mean ± s.d.). **e**–**j** Immunofluorescence of ECM (Collagen type 1, Collagen type 4, Fibronectin, Laminin) and myogenic (Myosin heavy chain, Actin, sarcomeric Actinin 2) proteins on decellularized scaffolds. **k** Coverage rate of ECM proteins detected through mass spectrometry analysis (*n* = 3). Scale bars: 50 μm.

Literature findings^19,39^ and our previous results^38^ demonstrated that a mixed population comprising myogenic progenitors and fibroblasts is the most suitable cell combination to promote myoblast migration and distribution, and completely repopulate a scaffold after cell injection. In this experimental setting, we used human skeletal muscle cells (hSkMC) expanded up to 10 passages and characterized by a good proliferation rate (KI67: 25.4 ± 5.8%), the expression of CD56 (96.4 ± 2.0%; Supplementary Fig. 1a) and myogenic markers (MYOD: 16.4 ± 7.7%; MYOG: 12.2 ± 6.8%), and differentiation capability (myogenic index: 83.9 ± 9.3%; Fig. 1a, b). Together with hSkMC, human fibroblasts (hFb), identified by the expression of TE7 (93.7 ± 8.4%) and alpha smooth muscle actin (αSMA, 61.5 ± 29.2%) markers (Fig. 1c) were used. As expected, hFb demonstrated negativity for the expression of CD56 (0.9 ± 0.1%; Supplementary Fig. 1b). These two populations were co-seeded at proportions of 85% hSkMC and 15% hFb, to recapitulate the physiological ratios obtained through enzymatic digestion of human muscle biopsies^40^. Co-culture experiments using standard 2D cultures highlighted the ability of cells to maintain this proportion unaltered during the log culture phase, suggesting a balanced proliferation between the two cell lines (Fig. 1d and Supplementary Fig. 1c).

As a biologic scaffold, decellularized mouse diaphragm ECM (dECM), prepared following a previously published protocol^41^, was used. As already shown, diaphragm dECM, together with the original architecture and myofiber structure, maintained major ECM components such as: Laminin (LAM), Collagen type 1 (COL1), and Collagen type 4 (COL4), but not Fibronectin (FBR); moreover, myogenic-specific proteins, such as Myosin heavy chain (MHC), muscle Actin (ACTA), and sarcomeric Actinin (ACTN2) were partially lost during the decellularization process (Fig. 1e–j and Supplementary Fig. 2a). These findings were confirmed by proteomic analysis that detected specific ECM components as comprising about 16% of a total of 66 discovered proteins; among these, Collagen was the most abundant protein (Fig. 1k). In addition, muscle fiber-specific proteins were found to still be present in dECM samples, with different isoforms of Myosin and Actin representing the largest proportions (Supplementary Fig. 2b, c; Supplementary Table 1).

### Bioreactor design and validation

**Fig. 2:**
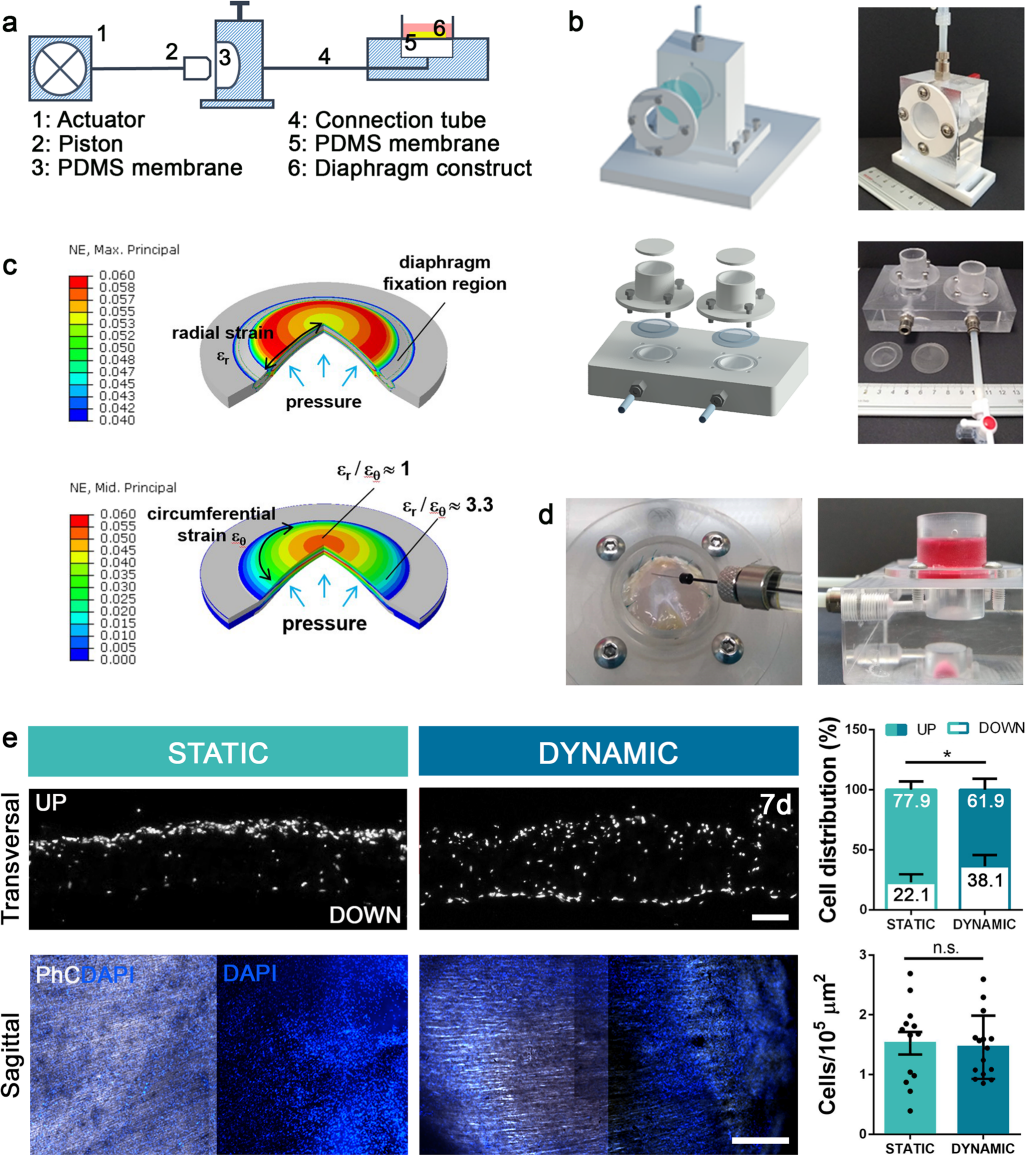
Bioreactor design, manufacture, and validation. **a** Simplified schematic of the bioreactor used: components 1–3 are part of the “motor bench unit” while components 4–6 are part of the “incubator bench unit”. **b** 3D-designed and manufactured bioreactor components. **c** FEM analysis was performed to evaluate the induced strain on PDMS membranes as a function of the hydrostatic pressure applied through the hydraulic circuit. Radial and circumferential strains are shown on a three-quarter section view. The diameter of the deformed membrane is 22 or 24 mm, according to the size of the stimulated diaphragmatic construct. **d** Representative images of cell injection and 3D culture. Scale bars: 1 cm. **e** Representative images of transversal and sagittal views of test 3D static and dynamic samples, with quantification of cell number (Cells/10^5^ μm^2^) and cell distribution (percentage of cells detected in the upper or lower scaffold side) (*n* = 3 per group; 10 random high-powered fields per sample; mean ± s.d.). PhC phase contrast, DAPI cell nuclei. Scale bars: 50 μm and 1 mm. *p < 0.05; n.s. not-significant.

Given the peculiar myofiber disposition in the diaphragm and the need to force cellular alignment to follow radial spatial orientation, a specific bioreactor system was designed and manufactured in-house. This bioreactor was developed considering the diaphragm’s shape, size, and physiological stretching. The bioreactor is based on a hydraulic circuit that deforms a series of thin and circular polydimethylsiloxane (PDMS) membranes, through the application of a controlled hydrostatic pressure. The PDMS membranes are the support of diaphragmatic constructs inducing a controlled stretch in the tissues. The hydraulic circuit is connected to electro-mechanical linear actuators regulated by a user-developed software. In this way, it is possible to modulate the magnitude and frequency of the applied pressure and consequent stretches of the diaphragmatic constructs fixed to PDMS membranes. Specific numerical models based on the finite-element method (FEM) were developed in the process of designing the bioreactor (Fig. 2a–c). Through these models, we defined the functions correlating the fluid volume change applied by the linear actuators and the radial strain induced on PDMS membranes that, in turn, induce a strain field to the fixed diaphragmatic constructs. FEM analysis of the interaction between PDMS membrane and diaphragmatic constructs (Supplementary Fig. 3) showed that, despite the generic shape and different stiffness of muscle and central tendon regions, the maximum principal strain induced in a large part of the muscle region was in the range of 4–6%, with a typical radial disposition of the principal strain direction. The strain vs. volume change functions were implemented in the software controlling the linear actuators. The maximum system capability tested with specific protocols showed that the bioreactor can apply mechanical stimuli corresponding to a radial strain up to 30% and frequency of 16 cycles per minute.

Literature findings highlighted that diverse mechanical stimuli (simple tensile strength, single or cyclic stimuli, different strain magnitudes) determine changes in the proliferation and maturation capacities of myoblasts and myotubes^42,43^. To validate the effect of radial mechanical strain on cell alignment and myotube formation on our bioreactor system, 2D culture experiments were carried out. Human SkMC were directly seeded on PDMS membranes and cultivated under both static and dynamic conditions inside the bioreactor (Supplementary Fig. 4). For dynamic samples only, a slow strain ramp (from 0% to 10%) was applied to avoid myotube detachment and mimic embryonal mechanical strain^44,45^. Through image analyses, dynamic samples showed coherency values and dominant direction of myotubes more similar to radial control images than did static cultures, which instead were analogous to the isotropic control. These results confirmed that radial mechanical stimulation influences myotube orientation and distribution.

Subsequently, to establish the best training protocol for the maturation of 3D diaphragmatic constructs, several magnitudes and stretch frequencies were tested on cardiotoxin (Ctx)-injured diaphragms cultivated in vitro inside the bioreactor for 2 days. After several attempts, this allowed us to exclude the conditions in which the cells did not survive or the tissue was excessively degraded (data not shown). The stimulation protocol with repeated cycles of 1 stretch/min and 5% mechanical strain was the dynamic condition that demonstrated a visible advantage in terms of morphological rescue of the damaged muscles compared with static culture (Supplementary Fig. 5). Injured diaphragms that underwent this dynamic stimulation protocol showed more rapid recovery of healthy morphometry than Ctx-injured tissues left in a static condition, regarding different analyzed variables, such as proportion of centrally nucleated myofibers or mean fiber cross-sectional area, achieving the same features as a fresh un-injured muscle.

Finally, to perform recellularization experiments, the hSkMC + hFb mixed population was injected into diaphragm dECM (Fig. 2d). From both macro- and microscopic perspectives, the test samples appeared markedly different from decellularized scaffolds and more similar to fresh diaphragms (Supplementary Fig. 6). The effects of dynamic culture and application of a mechanical stimulus were evident from the beginning, influencing transversal cell distribution inside the construct despite an equal number of cells compared with static culture. Indeed, in the dynamic samples a significantly higher percentage of cells was found in the lower construct side (38.1 ± 2.1%) than in the static culture (22.1 ± 3.5%; Fig. 2e) after 7 days.

### 3D diaphragmatic construct characterization

**Fig. 3:**
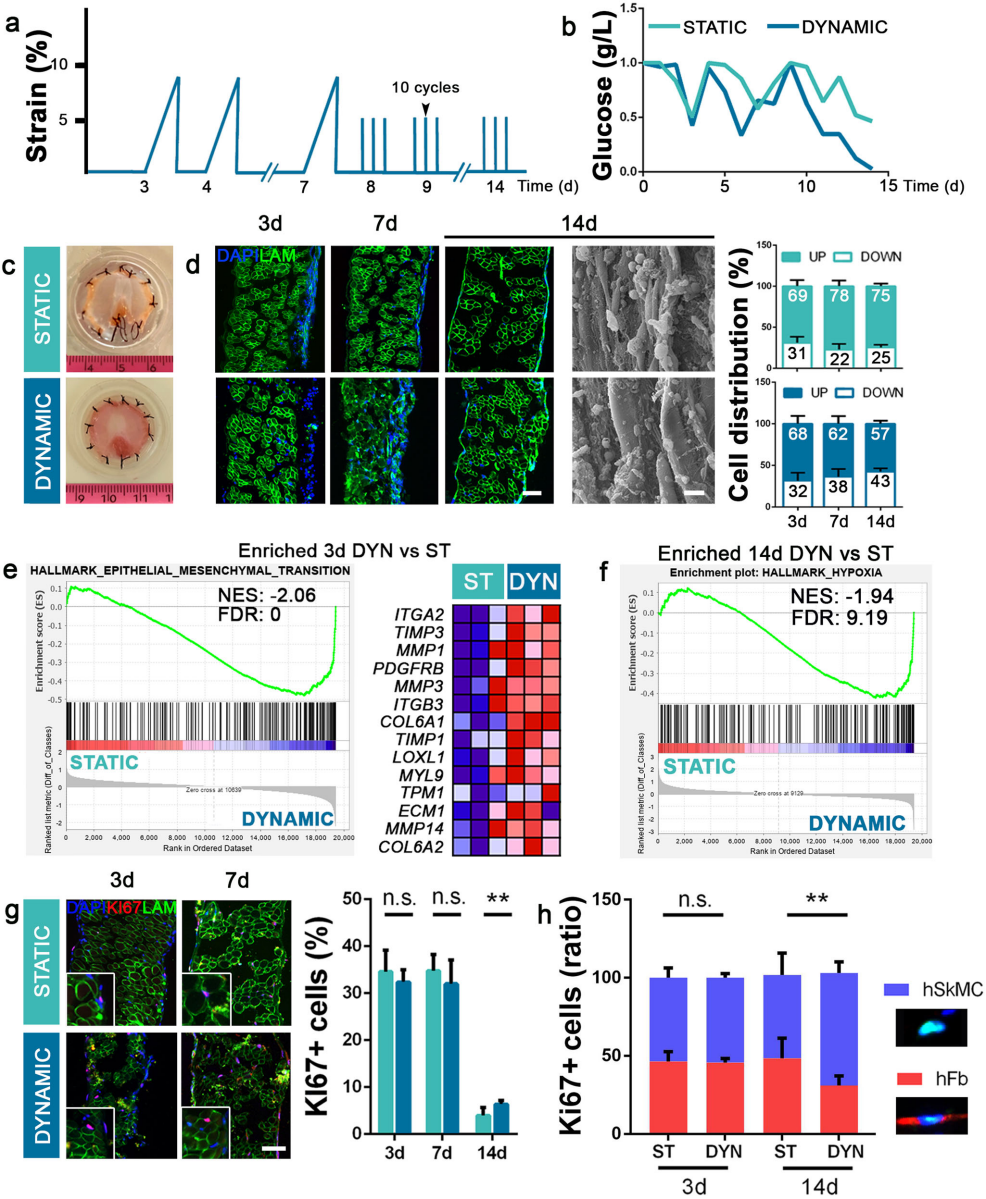
Diaphragm-like construct viability. **a** Dynamic mechanical stimulation protocol. **b** Glucose concentrations during 14 days culture were monitored using glucose-integrated sensors. **c** Gross appearance of static and dynamic samples after 14 days of culture. **d** Representative immunofluorescence and SEM images of static and dynamic constructs during the culture period. Quantification of cell distribution during the culture period in static and dynamic samples (UP and DOWN as indicated in the previous figure; mean ± s.d.). **e**, **f** Transcriptomic analysis: GSEA between static and dynamic samples at 3 days. Epithelial_Mesenchymal_Transition signaling pathway is positively enriched in 3-day-cultured dynamic samples; representatives among the top 20 enriched genes are shown (lower value in blue; higher value in red). Hypoxia signaling pathway is positively enriched in 14-day-cultured dynamic samples. **g** Representative immunofluorescence images and quantification of proliferating KI67 positive cells in static and dynamic samples (*n* = 3 per group for each time point; 10 random high-powered fields per sample; mean ± s.e.m.). **h** Ratio of proliferating hSkMC and hFb after 3 and 14 days of culture in both static (ST) and dynamic (DYN) samples (*n* = 3 per group for each time point; 10 random high-powered fields per sample; mean ± s.e.m.). Scale bars: 50 μm. *p < 0.05; **p < 0.01; n.s. not-significant.

Static and dynamic diaphragmatic constructs were maintained in culture for a maximum of 2 weeks, with analyses performed at 3, 7 and 14 days. Following our previously reported results and literature findings^42,43,46^, for dynamic cultures, a sequence of two different mechanical stimulation protocols was provided. Two days after cell seeding, the first type of stimulation was applied, using a slow ramp that went from 0% to 10% of maximum strain in 7 h, with a resting period of 17 h. From day 8, a training protocol was applied, in which a cycle of 10 stretches in 10 min (1 stretch/min) was provided three times a day, with maximum strain of 5% (Fig. 3a). Static and dynamic constructs were monitored throughout the culture period through integrated glucose sensors^47^, in order to follow glucose consumption and, indirectly, cell viability. Mean glucose consumption was similar between the culture conditions until day 3, after which the dynamic samples increased glucose metabolism day-by-day, in line with the physiological muscle response to training (Fig. 3b).

Both static and dynamic constructs demonstrated a healthy appearance and efficient recellularization throughout the tissue (Supplementary Fig. 7), without evidence of cell death (Supplementary Fig. 8a) and with the cells becoming more widely spread during the culture period, especially in the dynamic samples in which we found homogeneous cell repopulation at day 14 (Fig. 3c, d). SEM analyses performed at the last time point confirmed the efficient repopulation and healthy status in both culture conditions (Fig. 3d). Interestingly, during the tissue culture, we found an ever-greater percentage of cells in the lower side of the constructs only in dynamic samples, suggesting a continuous predisposition of cell to migrate when mechanically stimulated. This phenomenon is in line with the GSEA analysis, in which we discovered an enrichment in the epithelial-to-mesenchymal transition (EMT) signaling pathway in dynamic samples after 3 days of culture. Specifically, genes involved in ECM remodeling, such as different isoforms of *TIMP*, *MMP*, and *LOXL*, were strongly enriched in dynamic samples compared with the levels in those left in a static condition. At the same time, genes responsible for new ECM deposition, such as *COL6A1*, *COL6A2*, and *ECM1*, were found to be overexpressed, confirming the positive effect of mechanical stimulation in facilitating ECM remodeling and consequently triggering cell migration (Fig. 3e). In line with this, at the last time point, we detected enrichment of the hypoxia signaling in dynamic samples, indicating the less exposed and more sheltered cell position inside the scaffold, in which factors such as oxygen are less readily available (Fig. 3f).

The proliferation rate was high in all of the recellularized constructs (over 30%) until day 7, and then decreased with a slightly different pattern: static samples reached a mean of 2.2 ± 0.6% of KI67-positive cells after 14 days, whereas dynamic constructs showed a significantly higher percentage of proliferating cells (6.4 ± 0.8%) at the end of culture (Fig. 3g). Among all proliferating cells, at the beginning of the culture in both static and dynamic samples we observed equal percentage of KI67-positive cells between hSkMC and hFb. In contrast, after 2 weeks of culture, a significantly higher number of proliferating hSkMC were detected in the dynamic samples compared with the level in the static constructs (53.3 ± 14.0% static; 71.9 ± 7.1% dynamic; *p* < 0.05; Fig. 3h and Supplementary Fig. 8b).

### Fibroblasts and ECM interaction

**Fig. 4:**
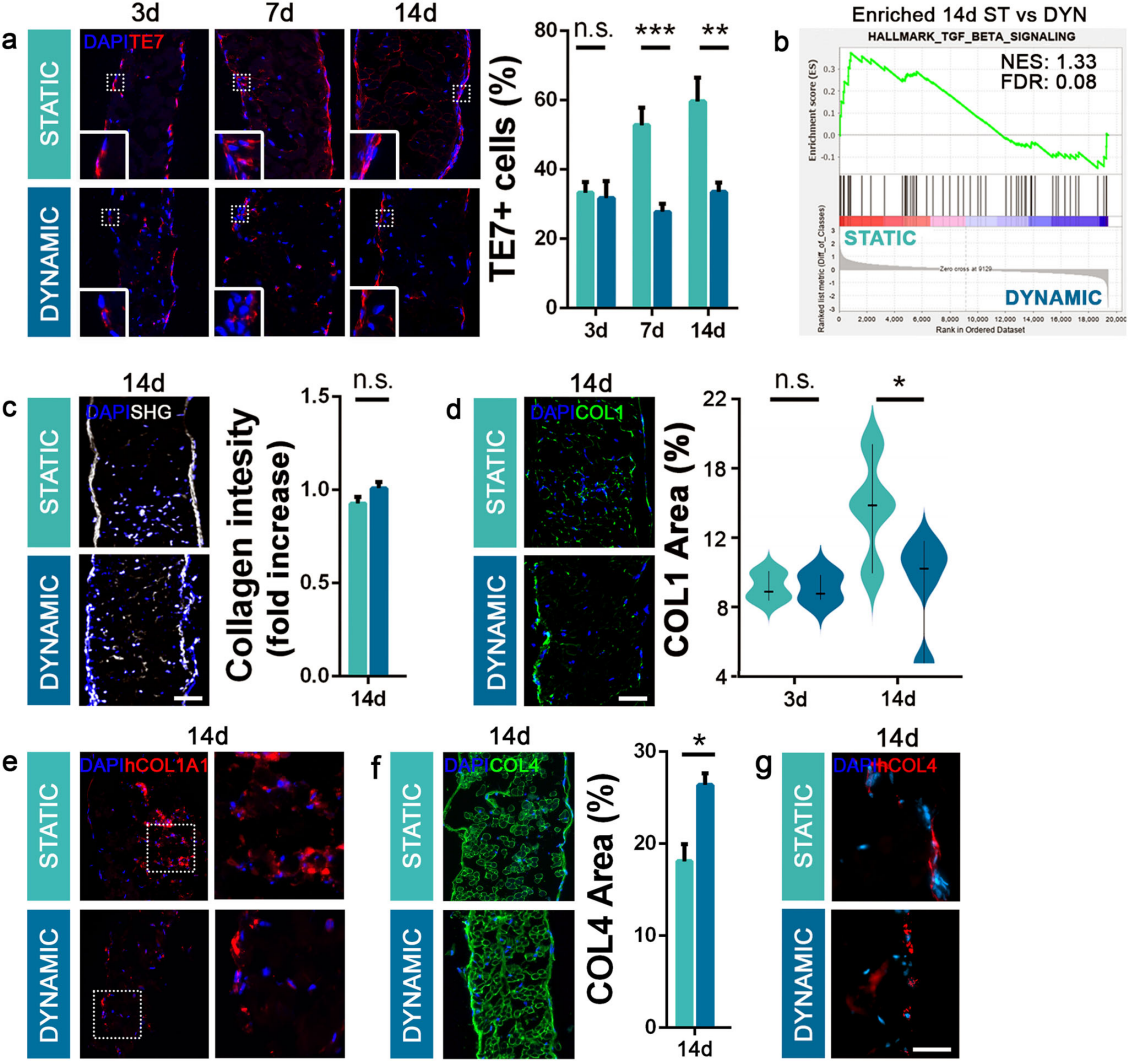
Diaphragmatic construct remodeling. **a** Representative immunofluorescence images and quantification of hFb (TE7-positive cells; 10 random high-powered fields per sample; mean ± s.e.m.) on static and dynamic samples during the culture period. **b** Transcriptomic analysis: TGF_beta signaling pathway is enriched in 14-day-cultured static samples. **c** Representative images of second harmonic generation (SHG) assay on static and dynamic samples after 14 days of culture (mean ± s.d.). **d** Representative images of Collagen type 1 on static and dynamic samples after 14 days of culture and quantification at 3 and 14 days (5 random high-powered fields per sample, mean ± s.e.m.). **e** Representative images of human-specific Collagen type 1 on static and dynamic samples after 14 days of culture. **f** Representative images of Collagen type 4 on static and dynamic samples after 14 days of culture and quantification at 14 days (5 random high-powered fields per sample, mean ± s.e.m.). **g** Representative images of human-specific Collagen type 4 on static and dynamic samples after 14 days of culture. For each assay, *n* = 3 per group per each time point. Scale bars: 100 μm. hCOL4 staining scale bar: 50 μm. *p < 0.05; **p < 0.01; ***p < 0.001; n.s. not-significant.

The proportions of hSkMC and hFb were monitored during the construct cultures. Although the same number of hFb were seeded in each condition, mechanical strain exerted a specific effect on dynamic samples influencing the cell proportion. In these constructs, the number of hFb remained around the physiological ratios in muscle tissue (33.5 ± 2.7%)^19,48^ from day 3 to 14, suggesting an active role of mechanical stimulation in controlling hFb proliferation. In contrast, such proliferation rapidly increased in static constructs, reaching about 60% of the total cell number after 14 days (59.7 ± 6.8%; Fig. 4a). From a molecular perspective, at the same time point, static samples were found to be enriched in the TGFβ signaling, consistent with the raised number of hFb detected in this condition (Fig. 4b).

With the SHG assay, which specifically detects Collagen types 1, 2 and 3^49^ (Fig. 4c), we did not identify a difference in the amount of Collagen between the two tissue cultures. Nevertheless, in the static condition, the increased proportion of hFb led to the continuous deposition of specific Collagen type 1, which covered a significantly higher muscle area after 14 days of culture (Fig. 4d) when compared with dynamic samples. This new deposition was confirmed by human-specific Collagen type 1 staining (Fig. 4e).

As shown in Fig. 1, Collagen type 4, which together with Laminin is responsible for muscle fiber basal lamina formation, was retained in the decellularized samples. Notably, after 14 days of culture, the total amount of COL4 increased in the dynamic samples compared with that of the static ones (Fig. 4f). This increase was due to the deposition of new protein, as evidenced by the presence of human-specific Collagen type 4 (hCOL4), highlighting the cooperation between seeded cells in the formation of basal lamina components (Fig. 4g)^50^.

### Myogenic differentiation

To investigate the molecular mechanisms underpinning the response of diaphragmatic constructs to the dynamic condition and confirm the effect of mechanical strain on construct differentiation and maturation, we analyzed several myogenic features. From the first days of stimulation, different hallmarks related to mechanical strain response and muscle cell metabolism, such as the oxidative phosphorylation signaling pathway, mTORC1, peroxisome and fatty acid metabolism^51^, were found to be enriched in dynamic constructs (Supplementary Fig. 9a).

**Fig. 5:**
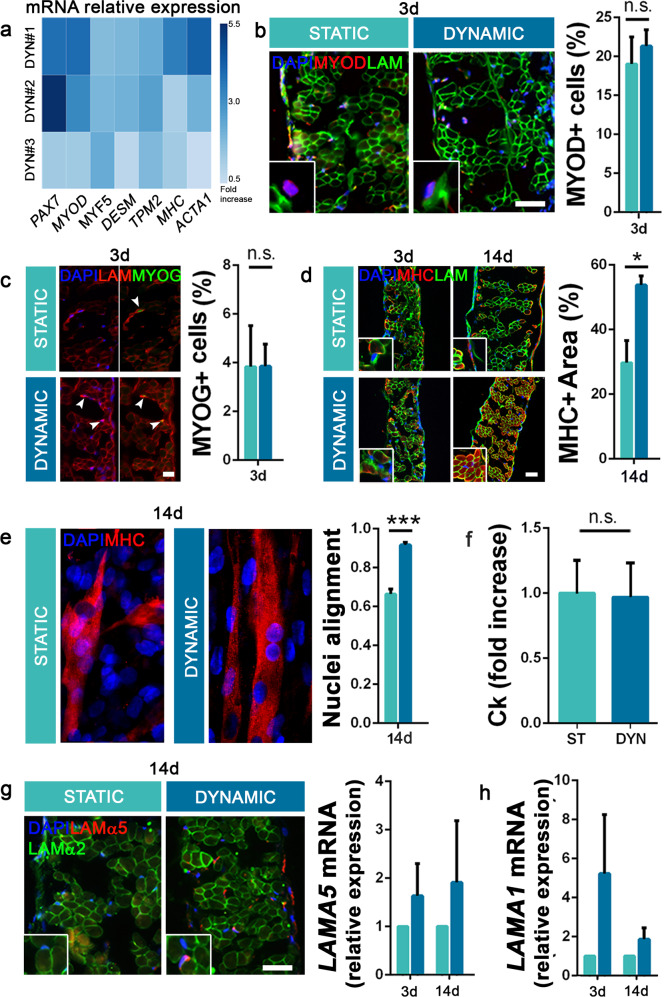
Diaphragmatic construct differentiation and maturation. **a** qRT-PCR analysis of early and late myogenic genes; data are expressed as mRNA expression of dynamic samples relative to that of static samples cultured for 3 days. **b**, **c** Representative immunofluorescence and quantification of MYOD-positive and MYOG-positive cells on 3-day-cultured static and dynamic constructs (10 random high-powered fields per sample). Scale bars: 50 μm. **d** Expression and quantification of MHC and Laminin in 3- and 14-day-cultured samples (5 random high-powered fields per sample; mean ± s.d.). **e** Representative confocal images of alignment of nuclei inside static and dynamic constructs, and relative calculation after 14 days of culture. **f** Quantification of Creatine kinase (Ck) released by static and dynamic samples after 14 days of culture (mean ± s.d.). **g** Staining and gene expression of Laminin-α5 on static and dynamic samples (mean ± s.e.m.). **h** qRT-PCR analysis for the expression of *Laminin-α1* in static and dynamic cultures (mean ± s.e.m.). Scale bars: 100 μm. For each assay, *n* = 3 per group per each time point. *p < 0.05; **p < 0.01; ***p < 0.001; n.s. not-significant.

With some expected differences among samples, the mechanical strain induced mean overexpression of both early (*PAX7, MYOD*, *MYF5*, and *DES*) and late (*TPM2, MHC*, and *ACTA*) myogenic genes in 3-day dynamic constructs compared with the level in the static condition (Fig. 5a). Muscle protein analysis evidenced an expected analogous number of MYOD-positive (19.0 ± 11.6% static; 21.3 ± 7.2% dynamic) and MYOG-positive (3.8 ± 3.8% static; 3.8 ± 3.2% dynamic) cells in both construct conditions at the beginning of the culture (Fig. 5b, c). Conversely, dynamic samples strongly expressed MHC-positive fibers at the end of mechanical stimulation (29.7 ± 6.8% of total tissue area in static; 53.8 ± 7.9% in dynamic after 14 days), consistent with the increased myogenic gene activation noted at an early time point (Fig. 5d). Moreover, only in dynamic samples, strong alignment among cell nuclei and myofibers throughout the construct’s thickness was observed, suggesting a pivotal role of mechanical strain in influencing 3D cell disposition (Fig. 5e, Supplementary Fig. 9b and Movie 1). This alignment is coherent with collagen orientation, which confirmed the generally better organization of dynamic cultured samples relative to their static counterparts (Supplementary Fig. 10). This occurred without showing tangible mechanical stress damage, given that similar levels of creatine kinase (Ck) were secreted in static and dynamic conditions (Fig. 5f). As previously observed^38^, the newly formed MHC-positive myofibers originated via different mechanisms, following the detected expression of embryonic laminin isoforms (together with mature Laminin-α2 isoform; Supplementary Fig. 9c). The presence and overexpression of Lamα5 in dynamic samples indicated that some of the cells integrated with the decellularized scaffold fibers, regenerating the pre-existing environment. At the same time, the higher transcription of *Lam*α*1* relative to that in static samples suggested that dynamic condition sustains myoblast activation, triggering the formation of new myofibers (Fig. 5g, h). Together with the identification of calcium transient response upon acetylcholine stimulation (Supplementary Fig. 11), these results indicate that our engineered strategy gave rise to a functional skeletal muscle tissue.

### In vivo 3D diaphragm construct application in established surgical mouse model of diaphragm defect

**Fig. 6:**
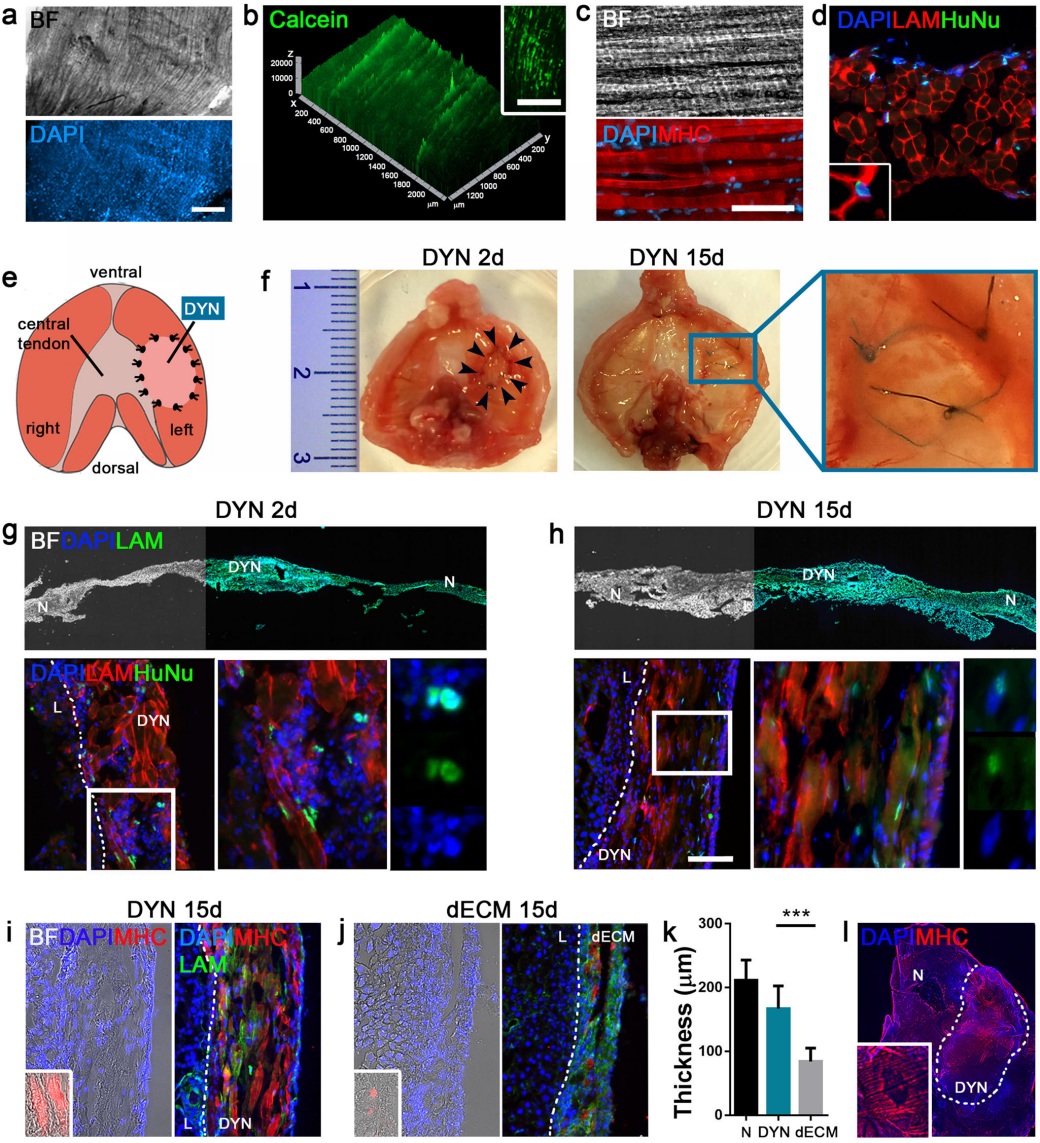
In vivo implantation of dynamic construct in CDH mouse model. **a** Representative images of implanted patch (BF: bright field). **b** Calcein staining to detect cell metabolic activity. **c** MHC staining of implanted construct. **d** Representative staining of human nuclei (HuNu) for the specific detection of human cells. **e** General scheme of surgically induced CDH mouse model (abdominal view of mouse diaphragm). **f** Gross appearance of treated diaphragms after 2 and 15 days of implantation (scale bar: 1 cm). **g**, **h** Representative images of treated diaphragms after 2 and 15 days (scale bar: 500 μm), with detection of HuNu-positive cells inside construct myofibers. Scale bars: 100 μm. **i**–**k** Representative images of DYN and dECM implants after 15 days, with quantification of implant thickness (*n* = 3 per group; 10 random high-powered fields per sample; mean ± s.d.). Scale bar: 100 μm; scale bar inlet: 50 μm. **l** Representative image of MHC expression on treated diaphragm. Scale bar: 500 μm. ***p < 0.001. L liver, N native diaphragm, DYN dynamic construct, dECM decellularized diaphragm.

To verify that the constructs generated in vitro can be efficient at supporting the surgical treatment of CDH, we implanted the dynamic samples after 14 days of culture into a well-established surgical model of diaphragmatic defect^52^. Before in vivo experiments, dynamic samples were validated for cell viability, metabolic activity (through Calcein staining), myogenic differentiation and maturation (Fig. 6a–d). Following the scheme shown in Fig. 6e, dynamic (or dECM) samples were transplanted into Rag2^−/−^γc^−/−^ mice and the grafts were analyzed after 2 and 15 days. As expected from the genetic mouse model background and our previously published results, the implanted constructs could integrate with the native diaphragms, to attract vessels and capillaries (Supplementary Fig. 12a), and to resist to mechanical strain exerted by the physiological muscle movements without developing liver herniation (Fig. 6f). Moreover, human nuclei (HuNu) were easily detectable inside the implants until sacrifice. Importantly, several HuNu were found still integrated into the construct myofibers, suggesting sustained and prolonged activity of human cells after in vivo implantation (Fig. 6g, h). One of the principal improvements of using a mature muscle construct compared with the ready-to-use acellular ECM is highlighted by the greater mean thickness of the recellularized implants (167.2 ± 9.1 μm DYN; 84.1 ± 5.4 μm dECM) that maintain size continuity with the rest of the native muscle (Fig. 6i–k and Supplementary Fig. 12b), and by the broad expression of MHC-positive fibers inside the construct also after 15 days of treatment (Fig. 6i, j, l).

## Discussion

Diaphragmatic muscle comprises right and left sides that are broadly symmetrical with some differences based on the presence of associated organs, including heart and lungs above and liver, stomach and spleen below. The muscle fibers in the two sternal regions of the diaphragm have a parallel orientation, whereas myofibers in the left and right costal regions have a radiating one^53^. This specific anatomy and myofibers disposition are well conserved among mammals, and are crucial for carrying out the main diaphragmatic functions. Injuries to the diaphragm, especially those in which a large muscle portion is missing as in CDH, are a major surgical problem, as the challenge is to close the defect while maintaining myofiber arrangement and tissue elasticity, which determine muscle functionality. Unfortunately, different approaches performed to date, involving either synthetic or biological substitutes, have not brought great improvements in the clinical treatment of CDH^35,37,54^, since they are unable to completely integrate with the native muscle and resist the incessant mechanical strain. In this work, we generated a diaphragmatic muscle using human cells in vitro and applied mechanical stimulation through a customized bioreactor platform. This provided a proof-of-concept of patient-specific muscle substitute production for treating large diaphragm defects.

For in vitro generation of the whole organ and to force cell alignment and widespread distribution following a radial orientation, a specific bioreactor was designed and developed, given that the classic and most widely used devices are generally conceived for in vitro linear muscle production^13,22,29^. Considering the different roles of static and cyclic mechanical stimuli, we established a dynamic culture protocol that provided a precise sequence of stimuli based on their impact on cellular behavior. We first stimulated the single cells seeded inside the scaffold with a slow and continuous strain that recapitulated embryonic bone elongation^24^, thus inducing myotubes formation and radial alignment within the dECM. Then, we applied repeated cyclic stimuli, to simulate what happens in the post-natal period in which training and physical exercise trigger muscle growth^55^. Mimicking the specific diaphragm environment as much as possible, we used a mixed population of hSkMC and hFb to engineer a humanized diaphragmatic dECM. Each of these cellular components is known to be important for cell maintenance and mutual conditioning within the tissue^19,39^.

In our experimental set-up, both static and dynamic samples demonstrated a healthy status throughout the culture period, with sustained proliferation for 7 days and constant metabolic activity highlighted by glucose consumption. However, it is important to underline how dynamic culture influenced the general cellular behaviors: the mechanical strain was effective at maintaining myogenic cell proliferation also after 14 days of culture; at the same time, glucose consumption was faster in cells grown under dynamic the condition, probably because, over the course of the culture, the massive maturation of myofibers resulted in increased glucose metabolism in response to mechanical stimuli^56^, suggesting stronger construct terminal differentiation. In skeletal muscle tissue engineering, fibroblasts are known to play a pivotal role in triggering myogenic cell migration^19,39^; in our samples this aspect was improved by the mechanical stimulus, which promoted better cell distribution throughout the scaffold thickness, a phenomenon that can also be partially supported by the increase in cell medium flow due to sample movements. The increased cell motility is underlined by transcriptomic analysis in which EMT signaling and ECM remodeling genes were found to be upregulated in dynamic samples. In skeletal muscle development, the EMT process gives rise to the long-range migration of cells, which retain an extensive mitotic capacity allowing their proliferation at target sites^57^. In our samples, the over expressed genes, induced *ECM1*, which encodes an anti-inflammatory protein that in other organs regulates T and B cells, modulates tissue homeostasis and inhibits latent TGFβ activation^58,59^. This supports the idea that mechanical stimulation increases regenerative capacity. Mechanical strain also influenced the ratio of hSkMC to hFb, which remained stable over the culture period only in dynamic samples. In static constructs, instead, the rate of hFb doubled after 2 weeks. Although fibroblasts make up a small proportion of cells in healthy skeletal muscle, they play an important role in maintaining muscle structure^60,61^; fibroblasts form a signaling niche necessary for long-term maintenance and proper cellular dynamics of the myogenic lineage^62^; thus, controlling their proliferation and general behavior allows better control over tissue culture. In this regard, in our constructs, the injected hFb spontaneously migrated towards the external areas of the diaphragmatic scaffolds, in a position that is reminiscent of the physiological situation, in which the epimysium, composed of connective tissue, is basically populated by fibroblasts^62^. Moreover, it was demonstrated in 2D cultures that mechanical strain suppresses hFb proliferation and cyclic stretch leads to a significant reduction in the level of Collagen type 1 deposited^63^. In line with these findings, we did not detect a difference in the total amount of Collagen between conditions, but clearly higher deposition of Collagen type 1 was found in static constructs relative to that in dynamic samples after 14 days, suggesting that static culture promotes the formation of a more fibrotic-like environment. The overexpression of ECM remodeling genes (e.g. *MMP1* and *MMP3*) after 3 days of dynamic culture suggests increased ECM degradation in mechanically stimulated samples, leading to a different change in the level of Collagen type 1 compared with that in the static culture. In addition, unstimulated samples displayed enrichment of the TGFβ signaling pathway after 14 days. TGFβ regulates the phenotype and function of fibroblasts and plays a role in myofibroblast trans-differentiation; it enhances the synthesis of matrix proteins such as Collagen type 1 and plays a critical role in the pathogenesis of many myopathies^64^.

From a myogenic perspective, the slow and gradual mechanical stimulation mimicked embryonal muscle development^65^. This stimulus is sensed by the cells that, from day 3 of culture, activated a series of transcripts in response to the applied strain. We found overexpression of the mTORC1 signaling pathway that is known as a key regulator in controlling skeletal muscle mass following contraction and mechanical load-induced hypertrophy^66,67^. Other molecular pathways specifically connected with myogenic regeneration were also found to be overexpressed, such as oxidative phosphorylation signaling, the main process involved in ATP production that is physiologically upregulated in response to an elevated energy demand. Moreover, several early and late myogenic genes were on average upregulated in dynamic samples compared with the levels in static ones, confirming that the mechanical stimulus exerts a specific action on muscle commitment even in 3D culture. The training protocol that we applied, instead, influenced myofiber maturation, as indicated by the strong expression of the MHC protein at day 14, and the high alignment of newly formed muscle fibers, together with the transcription of specific basal lamina components. The predominant laminin in healthy adult muscle fiber basal lamina is Laminin-α2-containing isoform (laminin-211). Generally, Laminin-α2 arises from the progressive replacement of the embryonic isoforms Laminin-α1 (laminin-111) and Laminin-α5 (laminin-511) upon remodeling events mediated by MMP^68^. In our dynamic samples, we found the overexpression of Laminin-α1 and Laminin-α5 relative to the levels in static constructs, suggesting stimulation of the production of embryonic basal lamina isoforms. Laminin-α1 does not display a preferential association with particular fates and is associated with activated, proliferating, as well as differentiating and self-renewing muscle progenitor cells^69^. Despite not reaching statistical significance, in dynamic samples we detected a downward trend in *LAMA1* transcription from 3 to 14 days of culture, which follows the reduction of single myoblasts and the maturation of diaphragmatic constructs. At the same time, in adult muscle Laminin-α5 is reported to overlay only regenerated myofibers^69^, and in this case we still detected high levels of *LAMA5* transcription after 14 days of dynamic culture together with the deposition of Laminin-α5 in many scaffold fibers. This suggests, as we previously demonstrated^38^, that once seeded inside diaphragm dECM, muscle cells can generate new skeletal muscle tissue through two different mechanisms: spontaneous fusion and integration with the decellularized fibers with partial regeneration of the pre-existing environment. These findings underline the usefulness of the bioreactor in broadly replicating muscle features and behavior. Thanks to the tunability of our system, by regulating frequencies and amplitudes of mechanical stimulation, it should be possible to simulate different types of muscle load and exercise, thus exploiting the bioreactor as a tool to analyze molecular and morphometric tissue responses in healthy and pathological conditions.

With the aim of overcoming the limitations of the currently available options for CDH treatment, we implanted the dynamic constructs in a mouse model of diaphragmatic hernia^52^, demonstrating that they are able not only to withstand continuous and incessant physiological strains without resulting in relapse or re-herniation, but can also easily integrate with the recipient tissue, reforming an intact and compact diaphragmatic muscle. Moreover, human cells were found to still be integrated in the muscle fibers of implanted constructs after 15 days of treatment, highlighting the active role of engineered cells in vivo for a prolonged period, and suggesting their potential utility in anticipated functionality. Despite the advantages presented by the application of acellular diaphragm dECM that we previously showed^41,52^ (easy manipulation and storage, no immune response, increased overall survival of treated mice compared with those with PTFE patch application), long-lasting resistance could not be guaranteed, since dECM implanted scaffold was demonstrated to be strongly remodeled and absorbed after 90 days of treatment. Despite this study being limited to only 2 weeks of in vivo transplantation, which was related to the difficulty of arranging long-term experiments in immunodeficient animals, we already noted a striking difference in the early remodeling of transplanted dynamic constructs when compared with acellular ECM. In this context, the generation and in vivo application of recellularized dynamic constructs represent an important step forward in validating the therapeutic potential of engineered diaphragmatic muscle. The reported findings lay the foundation for the scaled-up generation of customized diaphragmatic constructs starting from a piglet animal model^70^. After biological validation, this tool will help us to regenerate constructs with increased engraftment efficiency and strength, and reduce (even completely avoid) subsequent interventions that are frequently necessary to replace the graft. In a future scenario, clinically relevant constructs could be generated to overcome the major drawbacks of the current CDH surgical treatments. This new tissue engineering product may prove to be a significant opportunity as it would greatly increase the quality of life of little patients and their families.

## Methods

### Ethic statement

Diaphragm muscles were obtained from 3 months old C57BL/6j and Rag2^−/−^γc^−/−^ mice [Protocol Nos. 1103/2016 and 418/2020-PR approved by Animal wellness local ethics committee (Organismo per il Benessere Animale—OPBA, University of Padova and Fondazione Istituto di Ricerca Pediatrica Città della Speranza) and Italian Ministry of Health]; all in vivo experiments were performed using Rag2^−/−^γc^−/−^ mice in accordance with relevant guidelines and regulations.

### Cell cultures and maintenance

Primary normal human skeletal myoblasts (hSkMC, Gibco-Fischer Scientific) were grown in proliferative medium (PM) composed of DMEM low glucose (1 g/L d-glucose, Gibco-Fischer Scientific) with 20% fetal bovine serum (FBS; Gibco-Fisher Scientific), 10^−6^ M dexamethasone (Sigma-Aldrich), 10 ng/mL bFGF (R&D System), 10 μg/mL insulin (Gibco-Fisher Scientific) and 1% pen/strep (Gibco-Fisher Scientific). For myogenic differentiation, confluent hSkMC were cultured in fusion medium (FM), composed of αMEM (Gibco-Fisher Scientific) supplemented with 2% horse serum (HS; Gibco-Fisher Scientific), 10 μg/mL insulin and 1% pen/strep. Primary dermal fibroblast; normal, human, adult (hFb; ATCC PCS-201-012) were cultured in DMEM high glucose (4.5 g/L d-glucose, Gibco-Fisher Scientific) supplemented with 20% FBS, and 1% pen/strep. For co-culture experiments, the two populations were co-seeded in a ratio of 85% hSkMC and 15% hFb in 24-well plate. Co-cultures were maintained in PM for 2 days, and in FM for 2 days more. Cells were cultured at 37 °C, 5% CO_2_ with oxygen tension of 21% in humidified chamber.

### Cytofluorimetric analysis

Cell surface antigen expression was analyzed by flow cytometry on cells detached with trypsin–EDTA treatment at different passages. About 1 × 10^4^ hSkMC (*n* = 3), hFb (*n* = 3) or mixed cells (*n* = 3) were incubated with anti-human CD56-PE, 7-aminoactinomycinD was used as viability assay (both from BD Bioscience, Italy).

### Diaphragmatic muscle decellularization

Diaphragms were collected from mice, washed 2 times in 1× sterile phosphate buffered saline (PBS, Gibco-Fisher Scientific) and then transferred in deionized water in order to start the decellularization process. Diaphragms were processed with 3 detergent-enzymatic treatment (DET) cycles in order to obtain a complete cell removal. Each DET cycle was composed of deionized water at 4 °C for 24 h, 4% sodium deoxycholate (Sigma) at room temperature (RT) for 4 h, and 2000 Kunitz DNase-I (Sigma) in 1 M NaCl (Sigma) at RT for 3 h^41^. After decellularization, matrices were washed for at least 3 days in 1× PBS and immediately used or preserved in liquid nitrogen. For further information about decellularized diaphragm characterization see references^17,38,41,52^.

### Mass spectrometry

About 2.5 mg of three independent dECM samples have been resuspended in 0.2 mL of 50 mM ammonium bicarbonate buffer and digested following the protocol proposed by Naba et al.^71^. In brief, samples have been reduced, alkylated, deglycosylated (PNGaseF) and digested twice with Lys-C and Trypsin enzymes. Digested peptides were dried under vacuum and stored at −20 °C until analysis. Proteins identification: digested samples have been analyzed by a UHPLC-XEVO-G2-XS (Waters) mass spectrometer. The peptide mixtures were separated with a Biobasic C18 column, 5 μm, using a 3–45% linear gradient of CH3CN + 0.1% TFA (mobile phase B) in H_2_O + 0.1% TFA (mobile phase A) over at 110 min-long analysis. Mass spec data have been acquired in data-dependent mode in the 350–2000*m*/*z* mass range. Instrumental parameters were set as follow: source: ESI (+); precursor charge selection: from 2 to 4; resolution: 22,000. Data processing: mass spec data have been lock-mass corrected, peak picked, converted into mzML format and processed by Proteome Discoverer 2.2 (Thermo Fisher Scientific) using the Sequest HT algorithm for proteins identification. Search parameters were set as follow: database, UniprotKB reference proteome AUP000008227; enzyme, Trypsin (max. 2 missed cleavages); taxonomy, *Mus musculus*; precursor mass tolerance, 25 ppm; fragment mass tolerance, 0.08 Da. Fixed modifications: carbamidomethyl (C). Dynamic modifications: oxidation (M, P, K); deamidation (N, Q), and phosphorylation (S, T, Y). An acceptable proteins false discovery rate (FDR) was set <0.01 and a minimum of two non-redundant peptides were used for proteins identification. A total of 66 proteins were detected and listed in Supplementary Table 1.

### Bioreactor design, manufacturing, and validation

The bioreactor system is composed of two principal components: an incubator bench unit and a motor bench unit. In the incubator bench unit, the diaphragm is firmly placed upon the upper surface of an elastomeric membrane of polydimethylsiloxane (PDMS, Sylgard^®^ 184, Dow Corning). This membrane is fixed to close a reservoir of liquid (water) that can be changed in volume through a hydraulic circuit moved by an electro-mechanical linear actuator placed in the motor bench unit. The movement of the linear actuator can increase the liquid volume in the reservoir, deforming the membrane; the deformation of the elastomeric membrane induces in turn a deformation of the diaphragm that follows the membrane in its movements, being largely softer. The motor bench unit consists in a double crankshaft system, moving two indenters and deforming two separated hydraulic cameras, therefore generating a volume change into two distinct incubator bench units. Each pair of crankshafts is moved by a servomotor that, in turn, is connected to a Genuino board. The Genuino board is interfaced with a Personal Computer and controlled through a software specifically developed (MATLAB, MathWorks^®^). The software has a Graphical User Interface (GUI) that allow an easy programming of the mechanical cycles in terms of strain magnitude induced to the diaphragm tissue, frequency and overall time of application of the mechanical stimulus.

To design the bioreactor, we made use of FEM modeling. According to the radial symmetry of the systems in the incubator bench unit and a motor bench unit, we developed axialsymmetric models (Abaqus Unified FEA—SIMULIA™ di Dassault Systèmes^®^). The PDMS membranes were modeled as Neo-Hookean almost-incompressible material assuming an initial elastic modulus of 1 MPa. This datum was obtained from uniaxial tensile tests on rectangular strips of PDMS. The parts of the bioreactor made of polymethyl methacrylate (PMMA) were modeled as linear elastic and isotropic material with Young’s modulus of 3000 MPa and Poisson’s ratio of 0.2. Through nonlinear static analysis we obtained the functions between the change of volume induced by the indenter in the motor bench unit and the radial stretch induced in the PDMS membrane of incubator bench unit. We implemented this function in the software developed to control the Genuino board. The interaction between the PDMS membrane and the diaphragmatic construct was investigated through a specific FEM model including the shape of the diaphragm based on specific morphometric data. The mechanical properties for ECM and tendon were defined according to our previous published results^41,72^.

We obtained all the PMMA components of the bioreactor by using a numerical control cutter. The molds for the manufacturing of PDMS membranes were obtained by 3D printing. In the present configuration, the bioreactor is composed of three motor bench units that allow for using six incubator bench units simultaneously.

Different preliminary tests on the bioreactor were developed, applying high frequency mechanical cycles (up to 60 cycles/minute) for an overall time of five days, verifying the good reliability of the system. We developed also quasi-static tests to evaluate the correct deformation of the PDMS membrane of the incubator bench units, by measuring the amount of liquid volume moved by the membrane and comparing it with the expected liquid volume change in the reservoir as a function of the indenter displacement.

### Mechanical strain and myotube alignment in 2D cultures

About 1 × 10^5^ hSkMC were seeded on circular 22 mm PDMS membranes previously coated with 0.01% polydopamine for 1 h at RT under sterile condition in order to reduce the hydrophobicity of PDMS surface, and induce and enhance cell adhesion^73^. After 12 h, a slow ramp from 0% to 10% of mechanical strain was applied for 7 h, followed by a resting period of 17 h. After 4 days of culture, cells were fixed and analyzed for myotubes formation and alignment. To obtain a quantitative analysis of the image orientation we started with the weighed inner product:

1.1 $$\left\langle {f,g} \right\rangle \omega = \mathop {\iint}\limits_{R^2} {\omega (x;y)f(x;y)g(x;y){\mathrm {d}}x{\mathrm {d}}y}$$

where $\omega (x;y)$ ≥ 0 is a function that specifies the area of interest (usually represented by a square section of size *L* and centered in a target position (*x*_0_; *y*_0_)). Subsequently, the directional derivative along the unit vector $u_\theta = (\sin \theta ,\cos \theta )$ is considered

1.2 $$D_{u_\theta }f\left( {x,y} \right) = u_\theta ^{\mathrm {T}}\nabla f(x,y)$$

being $\nabla f = (f_x,f_y)$ the gradient of the image analyzed. The direction *u* is identified as that corresponding to the maximum derivative within the ROI

1.3 $$u = \arg \mathop {{\max }}\limits_{\left| {\left| u \right|} \right| = 1} |\left| {D_uf} \right||_w^2$$

1.4 $$\left\| {D_uf} \right\|_w^2 \,= \left\langle {u^{\mathrm {T}}\nabla f,\nabla f^{\mathrm {T}}u} \right\rangle _w \,= u^{\mathrm {T}}Ju$$

where

1.5 $$J = \left\langle {\nabla f,\nabla f^{\mathrm {T}}} \right\rangle _w = \left\lceil {\begin{array}{*{20}{c}} {\left\langle {f_x,f_x} \right\rangle _w} & {\left\langle {f_x,f_y} \right\rangle _w} \\ {\left\langle {f_x,f_y} \right\rangle _w} & {\left\langle {f_y,f_y} \right\rangle _w} \end{array}} \right\rceil$$

The so-called structure tensor *J* is a positive and symmetric 2 × 2 matrix. The main direction (1.3) is obtained by placing the derivative of $u^{\mathrm {T}}Ju + 1 - \frac{\lambda }{2}u^{\mathrm {T}}u$ respect to *u* equal to zero, thus obtaining the equation of the eigenvector: $Ju = \lambda u$.

This implies that the first eigenvector of *J* coincides with the dominant direction of the ROI and its eigenvalue corresponds to $\lambda _{{\mathrm {max}}} = {\mathrm {max}}\Vert D_uf\Vert _w^2$. On the contrary, the directional derivative is minimized in the orthogonal direction defined by the second eigenvector for which we have $\lambda _{{\mathrm {min}}} = {\mathrm {min}}\Vert D_uf\Vert _w^2$.

Therefore, the structure tensor contains all the relevant directional information. The parameters of interest are:

1. Orientation: $$\theta = \frac{1}{2}\arctan \left( {2\frac{{\left\langle {f_x,f_y} \right\rangle }}{{\left\langle {f_y,f_y} \right\rangle _w - \left\langle {f_x,f_x} \right\rangle _w}}} \right)$$
2. Coherency: $$C = \frac{{\lambda _{{\mathrm {max}}} - \lambda _{{\mathrm {min}}}}}{{\lambda _{\mathrm {{max}}} + \lambda _{\mathrm {{min}}}}} = \frac{{\sqrt {\left( {\left\langle {f_y,f_y} \right\rangle _w - \left\langle {f_x,f_x} \right\rangle _w} \right)^2 + 4\left\langle {f_x,f_y} \right\rangle _w} }}{{\left\langle {f_x,f_x} \right\rangle _w - \left\langle {f_y,f_y} \right\rangle _w}}$$

### Ex vivo mouse diaphragm injury and training

Diaphragmatic muscles obtained from 3 months old C57BL/6j mice [Protocol No. 1103/2016 approved by Animal wellness local ethics committee OPBA (Organismo per il Benessere Animale, Padova); all experiments were performed in accordance with relevant guidelines and regulations] were harvested and maintained in PM. Each sample was sutured on 22 mm circular PDMS membranes (sterilized with 70% industrial methylated spirits and left to evaporate under UV irradiation for 24 h prior to use) using 6-0 silk sutures (Ethicon). Cardiotoxin (Ctx, Latoxan, Portes-lés-Valence, France) was used to damage myofibers with a final concentration of 0.2 μM in PM. Ctx was administered for 6 h, then samples were immediately analyzed or maintained for 2 more days in PM following static or dynamic protocol, 3 cycles/day of 1 stretch/min for 10 min and 5% of mechanical strain.

### Diaphragm recellularization

hSkMC and hFb separately expanded for 3–10 passages were mixed in a ratio of 85 + 15%, respectively, and resuspended in 40 μL of 13% Collagen type 1 (Sigma-Aldrich), 10% Fibronectin (Sigma-Aldrich), 10% IGF-1 (ImmunoTools) in PM. The mix was injected at a density of 3 × 10^6^ total cell per scaffold directly inside the bioreactor chamber. Two hours from the injection, 2 mL of PM were added to the constructs, while further 3 mL were added after other 1.5 h. The PM was maintained for 4 days, and then changed according to the glucose consumption every other day before being replaced by FM for 3 days. PM was used for the rest of the culture period. The diaphragm-like constructs were analyzed after 3 (*n* = 7 static, 6 dynamic), 7 (*n* = 6 static, 9 dynamic) and 14 (*n* = 10 static, 19 dynamic) days of culture.

### Mechanical stimulation protocol

After cell injection, static and dynamic constructs were both cultured inside the bioreactor’s chambers. In the dynamic samples, two different mechanical stimulation protocols were applied:

1. *Slow stress ramp*: 48 h post injection, diaphragmatic constructs were stimulated with a slow ramp from 0% to 10% of mechanical strain for 7 h, followed by a resting period of 17 h. This stimulation protocol was applied for 5 days (from day 3 to day 7 of culture).
2. *Training protocol*: From day 8, a cyclic mechanical stimulation was applied with a protocol of 3 cycles/day of 1 stretch/min and 5% of mechanical strain for 10 min. This protocol was used for 7 days (from day 8 to day 14).

### Glucose consumption monitoring

An online glucose sensor (CITSens Bio Glucose Sensor, C-CIT Sensor) was integrated into each bioreactor well for real-time glucose measurement. The integrated sensor continuously measured the glucose in the culture media.

### Immunofluorescence

Tissue samples were fixed in 4% paraformaldehyde (PFA, Sigma-Aldrich) for 1 h at 4 °C. 8–10 μm thick sections were incubated with primary antibodies (1 h at 37 °C or overnight at 4 °C), washed and then incubated for 1 h at RT with labeled Alexa Fluor secondary antibodies, as listed in Supplementary table 2. Finally, nuclei were counterstained with fluorescent mounting medium plus 100 ng/mL 4′,6-diamidino-2-phenylindole (DAPI, Sigma-Aldrich). For each diaphragm, random pictures were collected with an inverted microscope. Fresh or decellularized diaphragms were used as controls.

For whole mounting immunofluorescence staining, fresh, dECM or recellularized mouse diaphragms were fixed in 4% PFA at 4 °C for 1 h. After rising in 1X PBS, the tissue was permeabilized and block for 1 h in Triton 0.5% X-100, containing 2% BSA and 4% goat serum. After two washing steps in 1X PBS, the sample was incubated overnight at 4 °C with primary antibodies diluted in 2% BSA. After primary antibody incubation, the sample was rinsed in 1× PBS and incubated for 1 h at RT with secondary antibodies. Tissue sample was counterstained with DAPI and pictures were obtain using ZEISS Axio Observer or ZEISS LSM800 confocal microscopy; analyses (image intensity and positive areas quantifications) were performed using ImageJ. Nuclei alignment was evaluated using the “Directionality” plugin in FIJI (https://imagej.net/plugins/directionality#fnref:1). This plugin has successfully been used to infer the preferred orientation of structures (in our case DAPI stained nuclei) present in the input images. Images in which there is a preferred orientation are expected to give a histogram with a peak then fitted with a gaussian curve^74^. The goodness of the fit ranges from 1 (good alignment) to 0 (bad alignment) and has been reported as “nuclei alignment”.

### Scanning electron microscopy (SEM)

Static and dynamic samples were rinsed with warm 1X PBS and fixed with 3% glutaraldehyde (Sigma) in 0.1 M phosphate buffer at room temperature overnight. After fixation and PBS washes, the samples were dehydrated in a graded ethanol–water series from 15% to 100% ethanol, critical point dried using CO_2_, and mounted on aluminum stubs using sticky carbon taps. Samples were mounted and coated with a thin layer of Au/Pd (approximately 2 nm thick) using a Gatan ion beam coater. Images were recorded with a JEOL JSM 6490 scanning electron microscopy.

### Second harmonic generation (SHG) analysis

The second harmonic generation (SHG) signal was detected using a custom-built multimodal microscope. The laser source used is a mode-locked Ti:Sapphire pulsed laser (Chameleon Ultra II, Coherent Inc.), with pulses of ∼140 fs at 80 MHz and tunable emission wavelength of 700 to 900 nm. The chosen excitation wavelength was 800 nm to detect the SHG signal (400 nm). The average laser power used was between 20 and 50 mW (10% of the maximum laser power). Images were acquired with a fixed resolution of 1024*1024 pixels and accumulation of 100 frames, with a pixel dwell time of 0.14 μs. All the SHG analyses were performed using Image-J as reviewed in Borile G et al. ^75^.

### Collagen coherency

Collagen coherency was calculated from SHG to verify the local dominant orientation of the fibers using OrientationJ, an ImageJ plugin^76^, as described by Rezakhaniha et al. ^77^. Coherency is bounded between 0 and 1, with 1 indicating areas with highly oriented structures and 0 indicating areas with randomly oriented structures.

### RNA extraction, microarray, and qRT-PCR analysis

Transcriptomic analysis was performed by comparing the global gene expression profiles of static and dynamic samples after 3 and 14 days of culture. Recellularized samples used for obtaining the gene expression profile were gently thawed in ice. Total RNA was extracted and quality checked, as reported in Trevisan C et al.^38^. Microarray analyses were performed using the Clariom S Assay (ThermoFisher Scientific), according to the manufacturer’s instructions, starting from 100 ng of total RNA. Data were normalized by R Bioconductor package (www.r-project.org) using Robust Multi-Array Average (RMA). Gene Set Enrichment Analysis (GSEA) was performed using GSEAv2.0 with gene ranked by differences of classes and statistical significance determined by 1000 permutations^78^. Gene set permutations were used to enable direct comparisons between static and dynamic samples. We used a cut-off of FDR < 0.1 to determine enriched gene sets. MSigDB derived from Hallmark curated database were select to perform enrichment. GSEA data were deposited using record GSE180636.

qRT-PCR analyses were performed using Platinum SYBR Green qPCR SuperMix-UDG (Invitrogen) and 2 μL of primers mix FW+REV (final concentration, 300/300 nM). mRNA relative expression was obtained by normalizing on housekeeping *β2-microglobulin*. Primer sequences are listed in Supplementary Table 3.

### Calcium transient analysis

At the defined ending time point (14 days), samples were allocated in petri dish and incubated with serum-free Dulbecco’s modified Eagle medium Low Glucose (DMEM, Thermo Fisher Scientific) supplemented with 20 μM of fluorescent calcium dye Fluo-4 AM (Thermo Fisher Scientific) and 0.04% PluronicTM F-127 (Thermo Fisher Scientific) at 37 °C for 30 min, as previously reported^38^. After incubation, samples were washed with DMEM. Live imaging analysis was performed using a fluorescent stereomicroscope (Leica M205 FCA) equipped with PLANAP0 1.0X objective. Video were recorded at 16 frame per second. Acetylcholine (Sigma-Aldrich) was reconstituted in PBS to produce a 100 mM stock solution and administered during the live imaging acquisition at the final working concentration of 10 μM. Calcium transient profiles were quantified within defined regions of interest by using Matlab 2021a (MathWorks) software. Each recorded trace was measured as relative changes in fluorescence emission intensity ΔF/F0, where F0 is the pre-stimulus basal fluorescence intensity at time 0 and ΔF is the normalized fluorescence intensity at time t.

### In vivo diaphragmatic hernia model

BALB/c Rag2^−/−^γc^−/−^ mice were operated in general inhalatory deep anesthesia after endotracheal intubation, following the protocol described in Trevisan et al. ^52^. Briefly, after a median superior incision and abdominal organs external dislocation, a 3 × 5 mm hole was surgically created in the left side of the native diaphragm. Afterwards the defect was closed by suturing either dECM (control) or 14 days recellularized dynamic constructs. Organs were then repositioned into the abdominal cavity; the abdominal wall was closed in two layers and the animals left to wake up under a heating lamp. Mice were euthanized by cervical dislocation at 2 (*n* = 3), and 15 (*n* = 3) days post-surgery. Implantation with dECM alone (*n* = 3) was used as control.

### Statistical analyses

Data are expressed as means ± s.e.m. or s.d. For immunofluorescence, at least 15 random high-power field areas were considered per analyzed sample. Using GraphPad Prism 6 v, statistical significance was determined with a two-sided parametric Student’s *t* test after D’Agostino–Pearson normality test, or non-parametric Kruskal–Wallis test. A *p*-value below 0.05 was considered statistically significant.

### Reporting summary

Further information on research design is available in the Nature Research Reporting Summary linked to this article.

## Supplementary information

Supplemental material

Supplementary Movie 1

REPORTING SUMMARY

## Data Availability

Data described in the manuscript are available from the corresponding authors on reasonable request. Microarray dataset was deposited in https://www.ncbi.nlm.nih.gov/geo/query/acc.cgi?acc=GSE180636.

## Code availability

Code used in the manuscript are available from the corresponding authors on reasonable request.
