# Supplementary material for: Customized bioreactor enables the production of 3D diaphragmatic constructs influencing matrix remodeling and fibroblast overgrowth
Source: NPJ Regen Med. 2022 Apr 25;7:25. doi: 10.1038/s41536-022-00222-x (PMC9038738; doi:10.1038/s41536-022-00222-x)
Supplement: Supplementary file 1 — Supplemental material [file 41536_2022_222_MOESM1_ESM.pdf]

Supplementary material

Customized bioreactor enables the production of 3D diaphragmatic constructs influencing matrix remodeling and fibroblast overgrowth

Edoardo Maghin[1,§], Eugenia Carraro[1,2,§], Daniele Boso[1,3], Arben Dedja[4], Mattia Giagante[1,3], Paola Caccin[2], Raluca Ana-Maria Barna[3], Silvia Bresolin[5], Alice Cani[5], Giulia Borile[6,7], Deborah Sandrin[6,7,8], Filippo Romanato[6,7,8], Francesca Cecchinato[9,10], Anna Urciuolo[8,9,10], Dorianna Sandonà[2], Paolo De Coppi[11,12], Piero G. Pavan[1,3,*] and Martina Piccoli[1,*]

[1] Tissue Engineering Lab, Fondazione Istituto di Ricerca Pediatrica Città della Speranza, Padova, Italy

[2] Department of Biomedical Sciences, University of Padova, Padova, Italy

[3] Department of Industrial Engineering, University of Padova, Padova, Italy

[4] Department of Cardiac, Thoracic and Vascular Sciences and Public Health, University of Padova, Padova, Italy

[5] Onco-Hematology Lab, Department of Women's and Children's Health, University of Padova, Padova, Italy

[6] Optics and Bioimaging Lab, Fondazione Istituto di Ricerca Pediatrica Città della Speranza, Padova, Italy

[7] Department of Physics and Astronomy, University of Padova, Padova, Italy

[8] L.I.F.E.L.A.B. Program, Consorzio per la Ricerca Sanitaria (CORIS), Veneto Region, Padova, Italy

[9] Neuromuscular Engineering Lab, Fondazione Istituto di Ricerca Pediatrica Città della Speranza, Padova, Italy

[10] Molecular Medicine Department, University of Padova, Padova, Italy

[11] Department of Specialist Neonatal and Pediatric Surgery, Great Ormond Street Hospital, London, UK

[12] Stem Cells and Regenerative Medicine Section, Department of Pediatric Surgery, UCL Great Ormond Street Institute of Child Health, London, UK

§ These Authors contributed equally to the work

* Correspondence should be addressed to: piero.pavan@unipd.it; m.piccoli@irpcds.org

Supplementary table 1. Protein list.

Accession	Description	Coverage [%]	# Peptides
Q99KI0	Aconitate hydratase, mitochondrial	4	2
Q3U122	Actin alpha 2, smooth muscle	44	11
P68134	Actin, alpha skeletal muscle	49	14
P60710	Actin, cytoplasmic 1	21	6
Q9CTH3	Actinin alpha 2	39	7
V9GXQ2	actin-like	14	2
P48962	ADP/ATP translocase 1	33	7
P51881	ADP/ATP translocase 2	26	5
A1BN54	Alpha actinin 1a	4	3
Q91VB8	Alpha globin 1	58	6
E9PUM3	Alpha-1,4 glucan phosphorylase	19	12
Q9JI91	Alpha-actinin-2	25	17
O88990	Alpha-actinin-3	8	6
P23927	Alpha-crystallin B chain	44	6
Q03265	ATP synthase subunit alpha, mitochondrial	24	10
P56480	ATP synthase subunit beta, mitochondrial	24	9
A2AKV0	ATP synthase subunit gamma, mitochondrial	22	2
P47857	ATP-dependent 6-phosphofructokinase, muscle type	7	4
Q8BFZ3	Beta-actin-like protein 2	11	3
A8DUK4	Beta-globin	40	5
P11087	Collagen alpha-1(I) chain	28	23
P28481	Collagen alpha-1(II) chain	2	2
P08121	Collagen alpha-1(III) chain	18	15
O88207	Collagen alpha-1(V) chain	2	2
Q04857	Collagen alpha-1(VI) chain	5	4
Q01149	Collagen alpha-2(I) chain	34	25
A0A087WS16	Collagen, type VI, alpha 3	2	3
Q6P8J7	Creatine kinase S-type, mitochondrial	12	3
P31001	Desmin	3	3
P62631	Elongation factor 1-alpha 2	11	4
Q6NY00	fructose-bisphosphate aldolase	29	7
Q9QZ83	gamma actin-like protein	18	5
A0A0A0MQF6	Glyceraldehyde-3-phosphate dehydrogenase	23	5
Q9WUB3	Glycogen phosphorylase, muscle form	20	14
P02089	Hemoglobin subunit beta-2	32	4
P62806	histone H4	21	2
P54071	Isocitrate dehydrogenase [NADP], mitochondrial	12	4
Q61789	Laminin subunit alpha-3	1	2
P08249	Malate dehydrogenase, mitochondrial	19	4
O55124	M-protein	3	3
Q6PCW9	MYL1 protein	19	3
B1AR69	Myosin- 13	14	29

P05977	Myosin light chain 1/3, skeletal muscle isoform	11	2
Q9CZ19	Myosin light chain 4	11	2
P97457	Myosin regulatory light chain 2, skeletal muscle isoform	31	4
Q5SX40	Myosin-1	51	96
G3UW82	Myosin-2	46	88
P13541	Myosin-3	21	40
Q5SX39	Myosin-4	47	86
Q02566	Myosin-6	32	58
Q91Z83	Myosin-7	41	73
P13542	Myosin-8	40	73
Q5XKE0	Myosin-binding protein C, fast-type	12	10
Q6P6L5	Myosin-binding protein C, slow-type	9	8
E9Q1W3	Nebulin	2	5
P52480	Pyruvate kinase PKM	6	2
Q8R429	Sarcoplasmic/endoplasmic reticulum calcium ATPase 1	21	18
O55143	Sarcoplasmic/endoplasmic reticulum calcium ATPase 2	9	7
E9Q8K5	Titin	8	191
Q8BSH3	Tropomyosin alpha-1 chain	20	6
D3Z6I8	Tropomyosin alpha-3 chain	8	2
P58774	Tropomyosin beta chain	26	7
F6QC77	Troponin I, slow skeletal muscle	14	2
Q60932	Voltage-dependent anion-selective channel protein 1	25	5
G3UX26	Voltage-dependent anion-selective channel protein 2	11	2
J3QMG3	Voltage-dependent anion-selective channel protein 3	12	3

Supplementary table 2. Antibodies' list.

Antibody	Dilution	Manufacturer
αSMA (Mouse)	1:100	Abcam
Muscle Actin AF488	1:100	Invitrogen
Alfa Sarcomeric Actinin 2 (Mouse)	1:100	Sigma-Aldrich
Fibronectin (Rabbit)	1:100	Abcam
Human Nuclei (Mouse)	1:200	Millipore
KI67 (Rabbit)	1:100	Abcam
Laminin (Rabbit)	1:200	Sigma-Aldrich
Laminin α2 (Rat)	1:100	Sigma-Aldrich
Laminin α5 (Mouse)	1:100	Termo Fisher
MHC (Mouse)	1:100	R&D Systems
MYOD (Mouse)	1:50	Dako
Myogenin AF488	1:80	Invitrogen
TE7 (Mouse)	1:100	Millipore
Type 1 Collagen (Rabbit)	1:100	Abcam
Type 4 Collagen (Rabbit)	1:100	Abcam
Human-specific Type 1 Collagen	1:100	Sigma-Merk
Human-specific Type 4 Collagen AF647 (mouse)	1:100	Invitrogen
Anti-Rabbit 488	1:200	Life Technologies
Anti-Rat 488	1:200	Life Technologies
Anti-Rabbit 594	1:200	Life Technologies
Anti-Mouse 594	1:200	Life Technologies
Anti-Rat 568	1:200	Life Technologies

Supplementary table 3. Primers' list.

Gene	NM_	Sequence	Amplicon (bp)
PAX7	002584.2	Fw: 5'-TAGCCGCGTGCTCAGAATCA-3'	133
		Rev: 5'-GTCCAGCCGGTTCCCTTTGT-3'	
MYOD	002478.4	Fw: 5'-CAGCCCGCGCTCCAACTGCT-3'	126
		Rev: 5'-TTCCCGGGCCTGGGTTCGCT-3'	
MYF5	005593.2	Fw: 5'-CCACCTCCAACTGCTCTGAT-3'	171
		Rev: 5'-AGGTGATCCGGTCCACTATG-3'	
DESM	001927.3	Fw: 5'-TTCCGAGAAACCAGCCCTGA-3'	101
		Rev: 5'-TCACTGACGACCTCCCCATC-3'	
MHC	005963.3	Fw: 5'-CAGCCTGGAGCAGCTGTGCAT-3'	176
		Rev: 5'-TGCCCATAGGCTTCTCGATGAGCTC-3'	
ACTA1	001100.4	Fw: 5'-CCCGCCCAGAAACTAGACACA-3'	134
		Rev: 5'-CCCACGATGGACGGCAACAC -3'	
TPM2	213674.1	Fw: 5'-CCCGAGCAGAGTTTGCCGA-3'	126
		Rev: 5'-GCAGGGTCTGGTCCAAGGTCT-3'	
LAMA1	005559.3	Fw: 5'- GATGGCAACAGCGCAAACCC -3'	196
		Rev: 5'- AGTTTCCAGGTCGAGGGGCA -3'	
LAMA5	005560.4	Fw: 5'-GTGCCCGGTGCCTACAACTT -3'	112
		Rev: 5'-CCCGGCACATACAGGGAACC -3'	
β2M	004048.2	Fw: 5'-CAACCTCCAACTGCTCTGAT-3'	161
		Rev: 5'-GGTGTGCTCGCGCTACTCT-3'	

Supplementary figure 1. Flow cytometry of CD56 expression in hSkMC, hFb and mixed population.

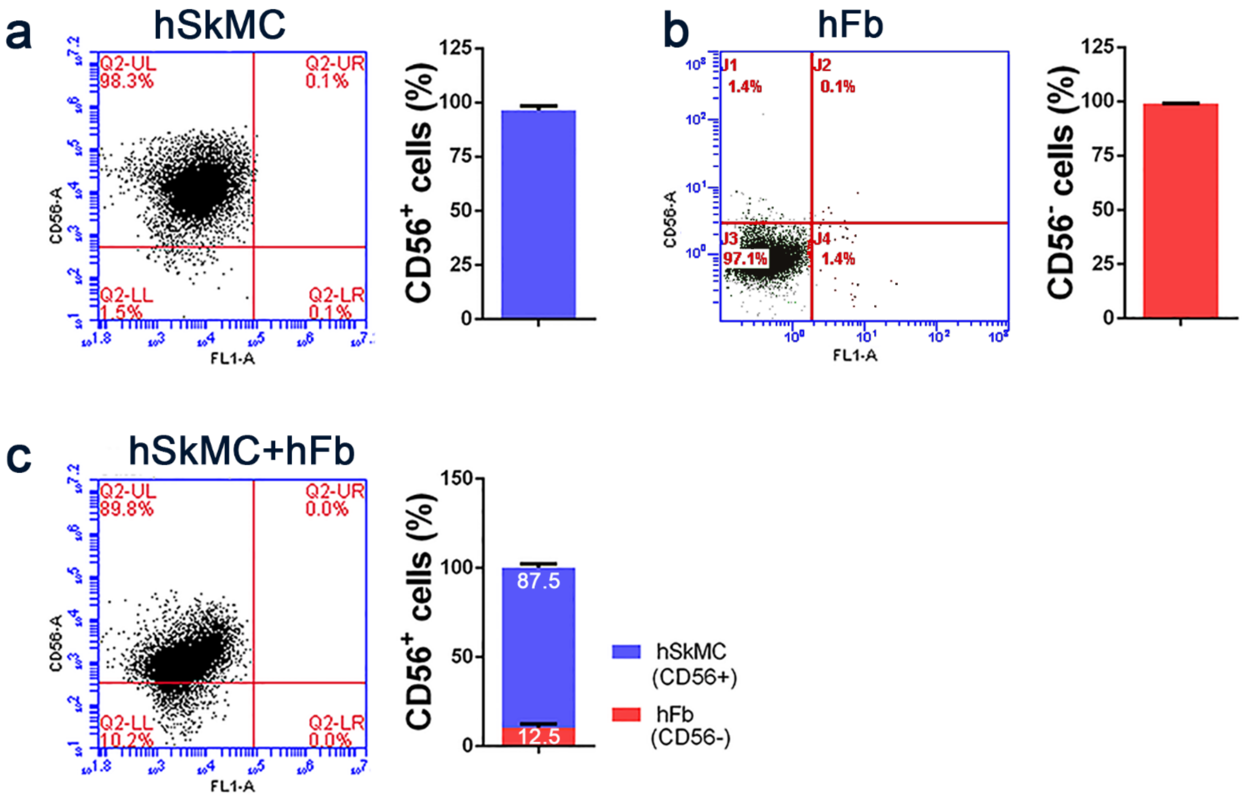

a-b. Human SkMC and hFb were stained with antibody anti CD56-PE; more than 95% of hSkMC resulted positive after flow cytometry investigation, whereas hFb did not expressed the marker ($CD56^-$: 99%). **c.** Four days after 2D culture, the seeded mixed population maintained the proportion of 85% of hSkMC ($CD56^+$) and 15% of hFb ($CD56^-$). Data are expressed as mean±s.d.

Supplementary figure 2. Decellularized ECM characterization.

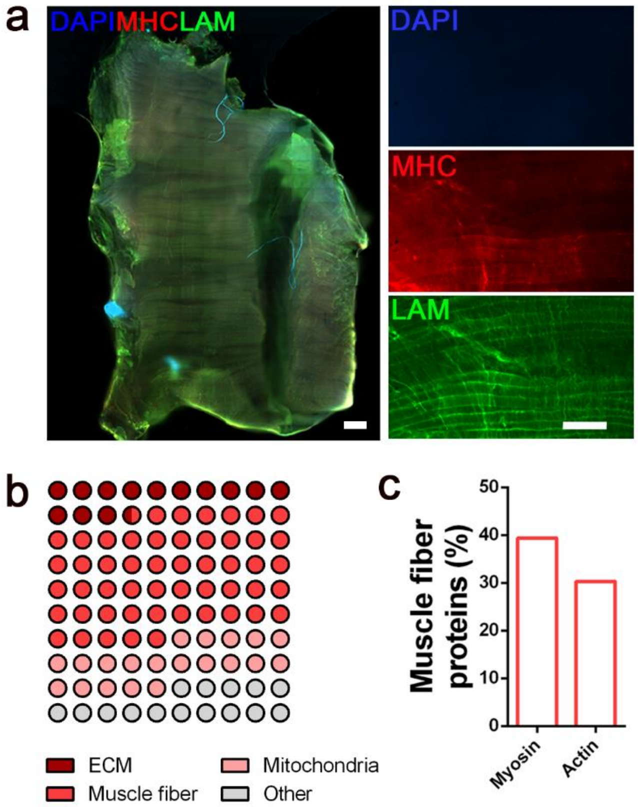

a. Decellularized hemidiaphragm whole mount staining. **b.** Schematic representation of 66 discovered proteins through mass spectrometry with relative abundance (ECM: 13.6%; Muscle fiber: 51.5%; Mitochondria: 19.7%; Other: 15.2%). **c.** Relative abundance of different myosin and actin isoforms inside muscle fiber group. Scale bars: 100 μm.

Supplementary figure 3. Finite element model analysis of PDMS membrane and diaphragm deformations.

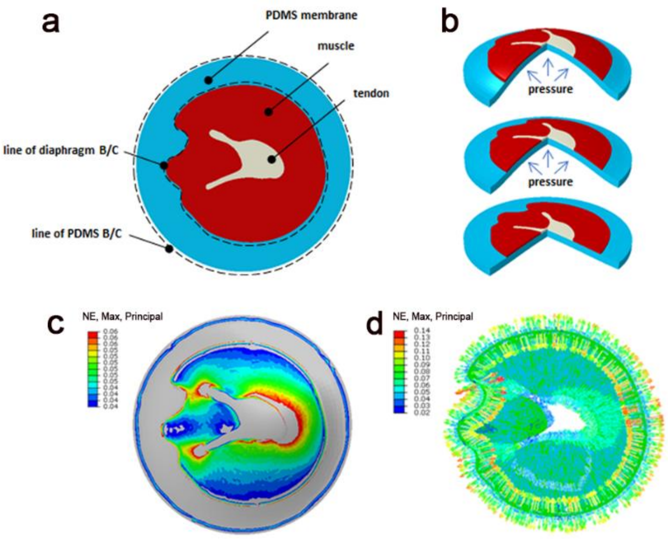

a. Geometry of FEM model including PDMS membranes and diaphragm. Dotted lines represent the region of Boundary Conditions (B/C) of the external ring of PDMS membrane and that of diaphragm muscle to PDMS through the rib. **b.** Sequence of PDMS membrane and diaphragm deformations with the increasing of hydrostatic pressure in the lower side of the PDMS membrane. **c.** Field of maximum principal strains: the coloured regions present strain included between 4% and 6%. **d.** Field of the maximum principal strain directions in the diaphragm, showing the typical radial alignment.

Supplementary figure 4. Radial mechanical strain effect on myotube disposition and orientation.

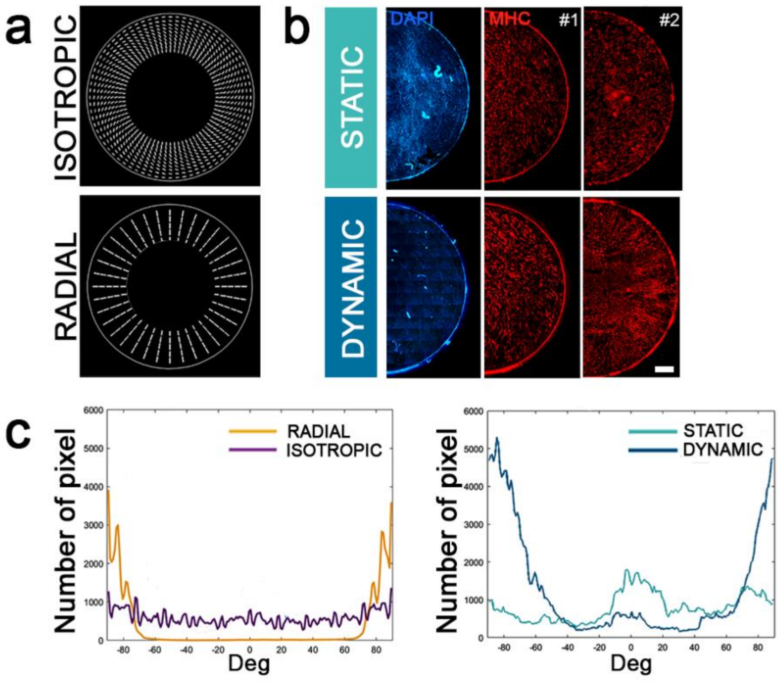

a. *Ad hoc* designed images as negative (isotropic) and positive (radial) control of myotubes' alignment. **b.** Representative pictures of static and dynamic 2D cultures with nuclei stained with DAPI and myotubes with MHC (#1: sample 1; #2: sample 2). Scale bar: 2000 μm. **c.** Representative histograms of myotube dominant direction on control and sample images. The angles ±90° represent the local radial direction of the sample. Higher values of the curves correspond to a predominant spatial orientation of the single regions (myotube) along the direction identified by the corresponding angle. From the graphs it is evident that the static samples have a generic myotubes' spatial alignment, similar to the isotropic control distribution, while the dynamic ones show a spatial alignment pattern that mimics the radial control.

Supplementary figure 5. Dynamic training protocol set up.

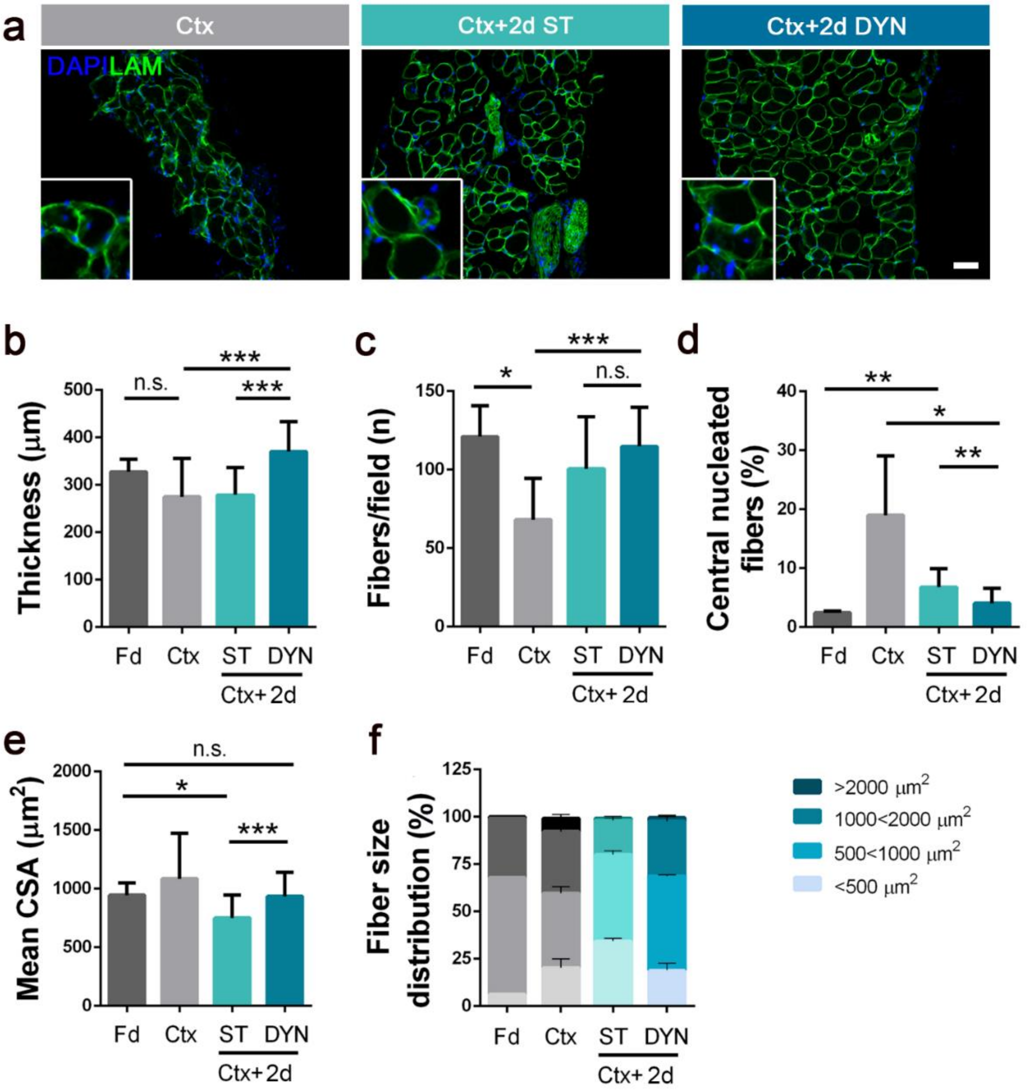

a. Representative images of diaphragm samples conditions: Ctx= diaphragm treated with cardiotoxin for 6 hours; Ctx+2d ST= diaphragm treated with cardiotoxin for 6 hours and left in static condition for 2 days; Ctx+2d DYN= diaphragm treated with cardiotoxin for 6 hours and mechanically stimulated for 2 days. Scale bar: 50 μm. **b-f.** Morphometric analyses performed on fresh diaphragm (Fd) and Ctx treated diaphragm (Ctx, Ctx+2d ST, Ctx+2d DYN; n=3 per each group). CSA= cross sectional area; data are expressed as mean±s.d.

Supplementary figure 6. Fresh, recellularized and decellularized samples comparison.

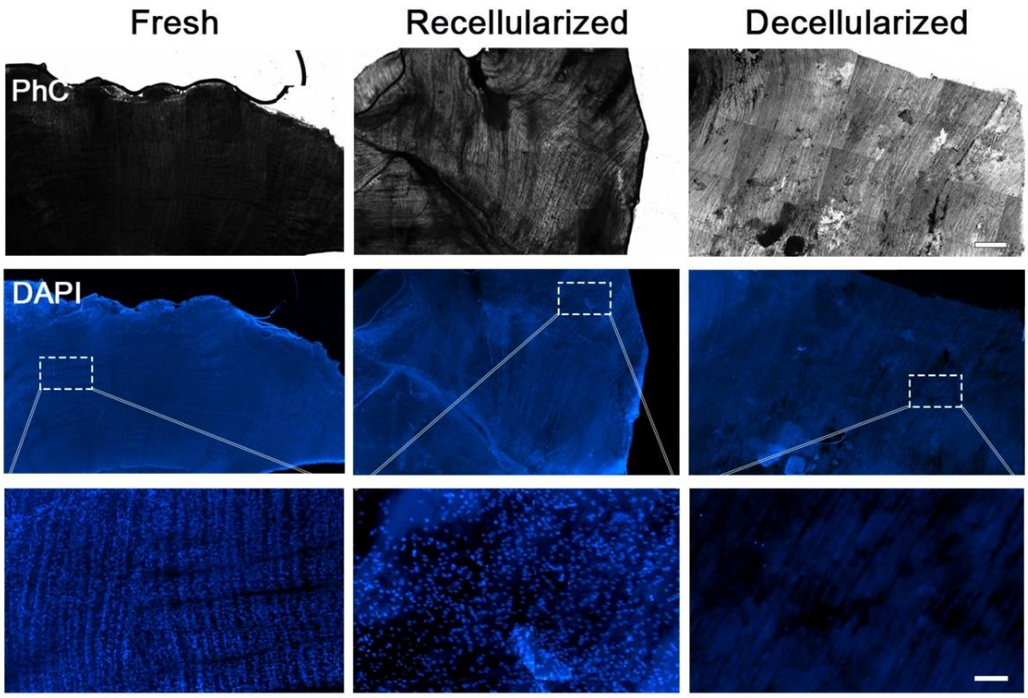

Macroscopic (PhC) and microscopic (DAPI) comparison among fresh, recellularized and decellularized diaphragms in terms of cell content (nuclei are stained with DAPI) and distribution. Scale bar: 100 μm.

Supplementary figure 7. Macroscopic view of representative slides of static and dynamic samples after 3, 7 and 14 days of culture.

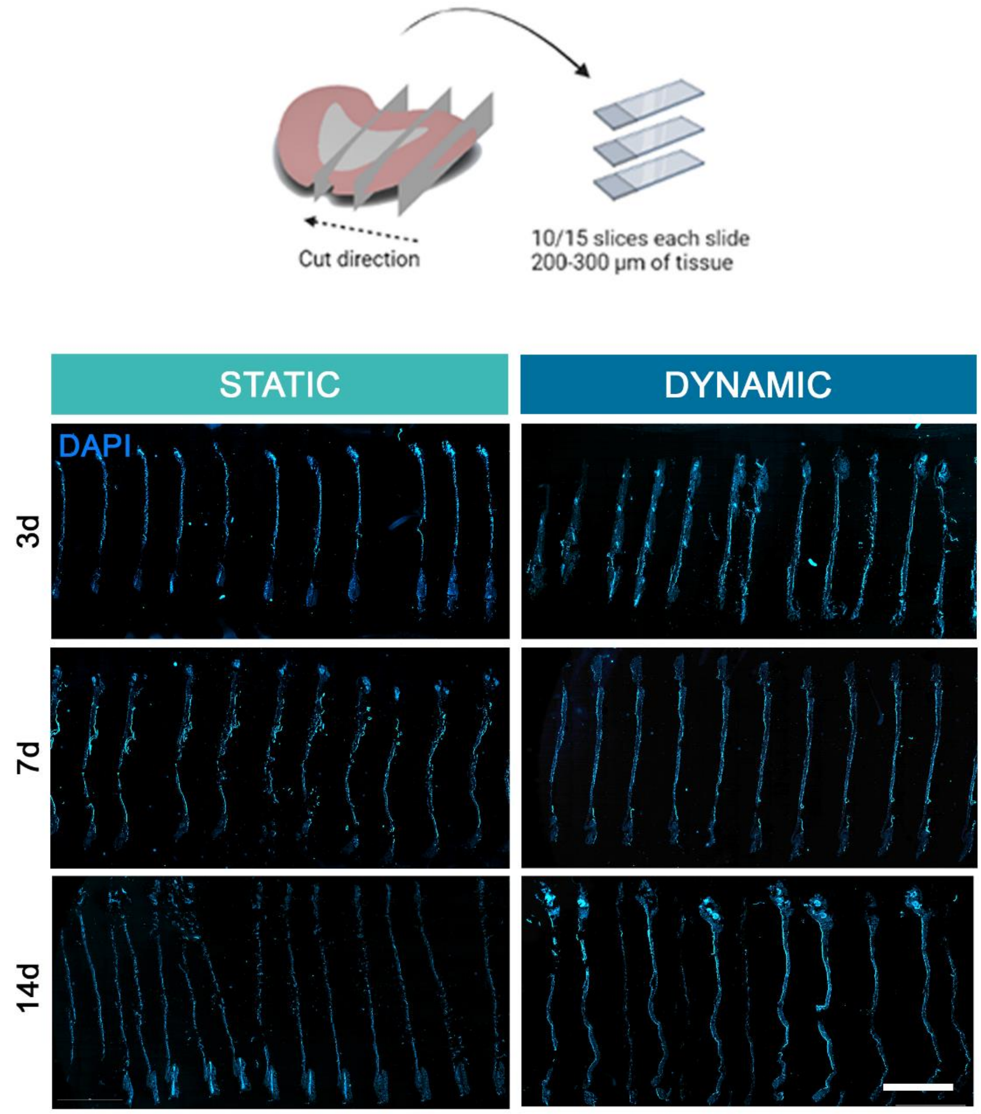

Scheme of diaphragmatic construct sectioning and representative images of static and dynamic sample sections taken in different construct areas: cells are uniformly distributed throughout tissue. Scale bar: 5000 µm.

Supplementary figure 8. Staining for cell death detection and proliferation during tissue-like construct cultures.

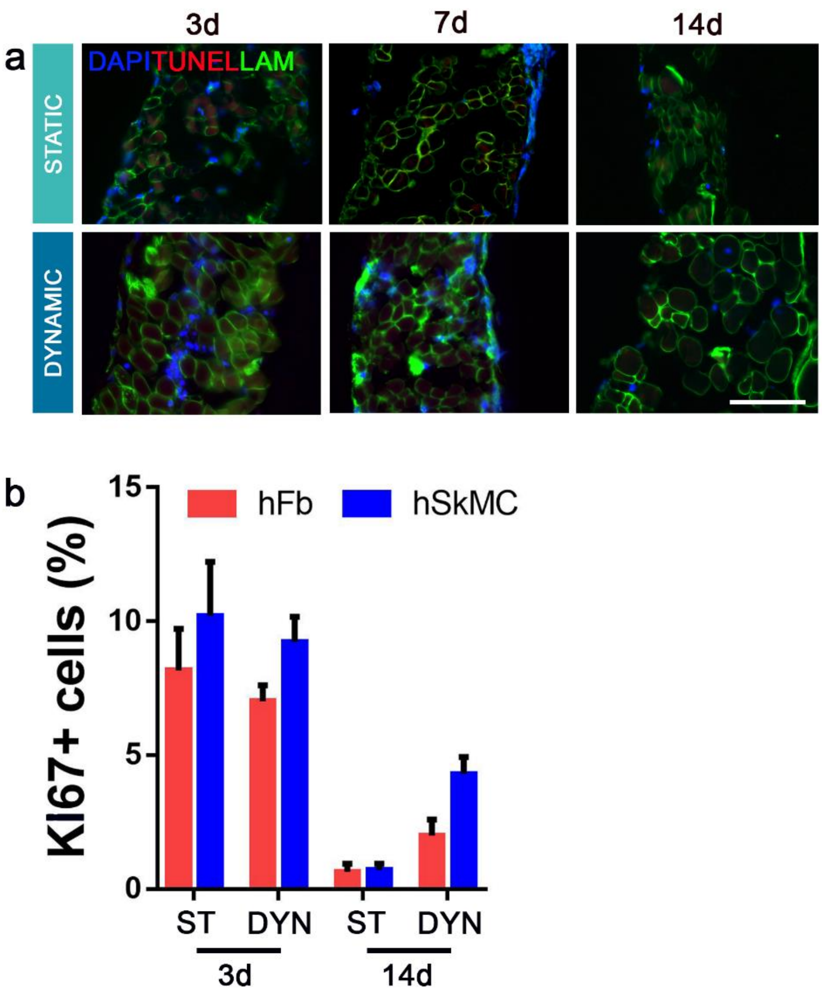

a. Representative images of TUNEL staining on different time point of static and dynamic cultured samples (n=2 per group each time point). **b.** Distinct quantification of proliferating (KI67+) hFb and hSkMC after 3 and 14 days of static and dynamic cultures (n=3 per sample; mean ± s.d.). Scale bar: 100 µm.

Supplementary figure 9. Transcriptomic analysis and myogenic differentiation.

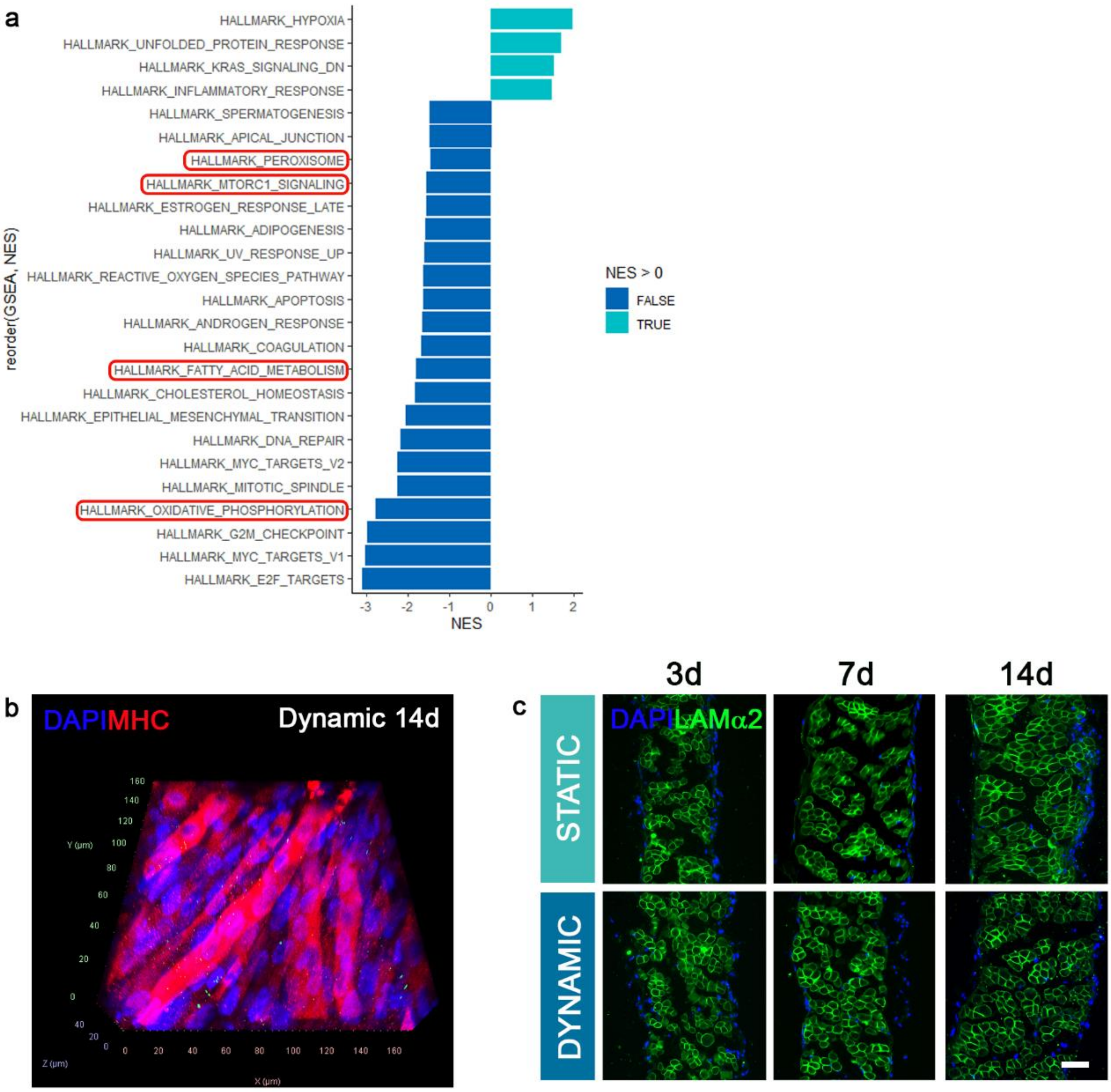

a. Enriched pathways in dynamic respect to static samples after 3 days of culture. Highlighted hallmarks (oxidative phosphorylation, fatty acid metabolism, mTORC1 signaling, and peroxisome) are directly involved in skeletal muscle metabolism. NES: Normalized Enrichment Score. **b.** 3D reconstruction of a representative dynamic sample after 14 days of culture. **c.** Expression of Laminin-α2 in static and dynamic samples over time. Scale bar: 50 μm.

Supplementary figure 10. Coherency values and goodness of static and dynamic cultured samples.

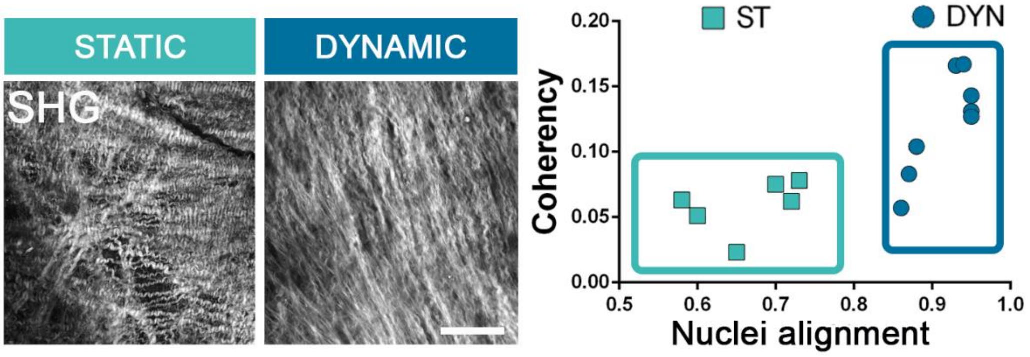

Comparing the nuclei alignment analysis and the coherency values (determined from the orientation of collagen fibers), the general organization and alignment of the structures can be defined. Dynamic constructs demonstrated a better organization than static samples after 14 days of culture (n=2 samples per condition). Scale bar: 100 μm.

Supplementary figure 11. ***In vitro*** **diaphragmatic construct functional analysis.**

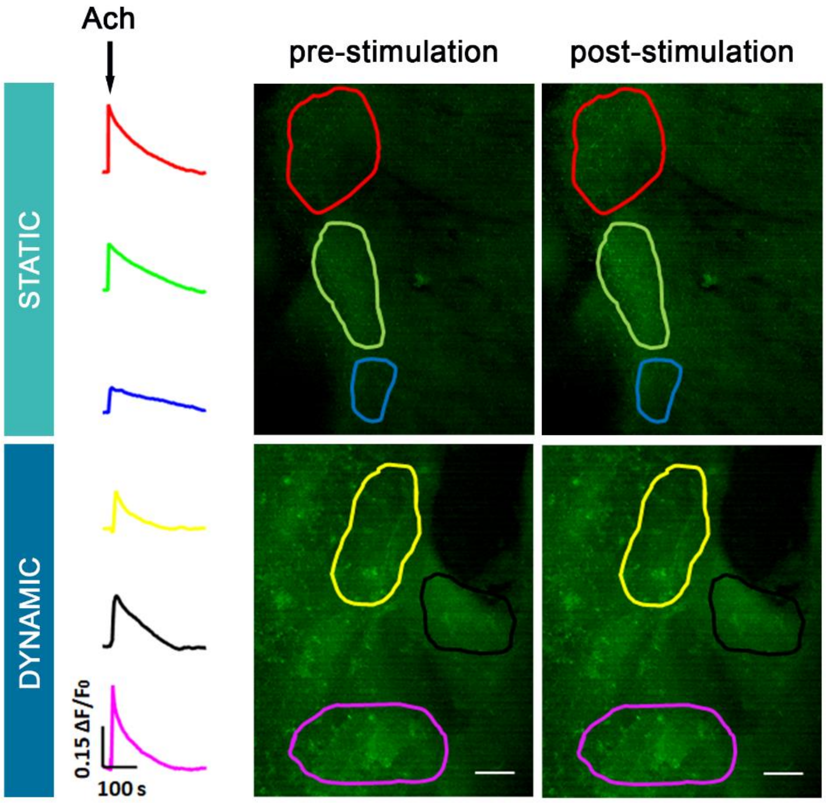

Representative images of static and dynamic samples incubated with Fluo4 (pre-stimulation) and stimulated with acetylcholine (post-stimulation). After stimulation, calcium transients were quantified in different regions of interest. Each colored trace ($\Delta F/F_0$) corresponds to the same colored region. Scale bar: 500 μm.

Supplementary figure 12. Revascularization of implanted dynamic constructs.

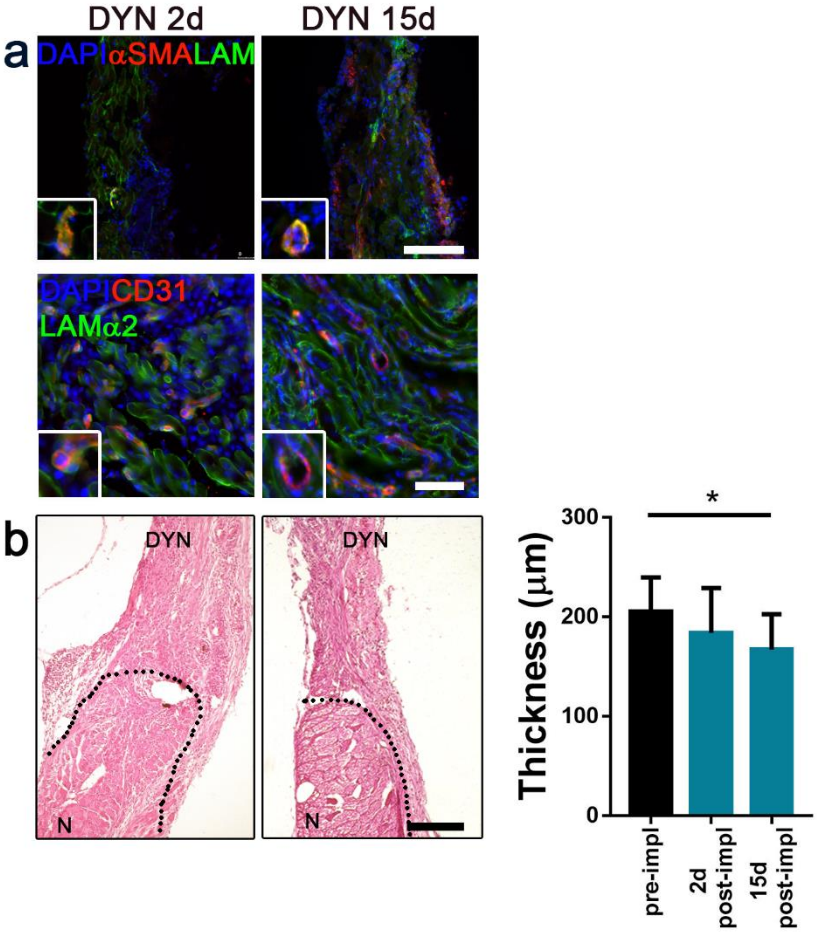

a. Representative images of αSMA and CD31 staining in *in vivo* implanted dynamic constructs after 2 and 15 days. Inside implanted construct are visible vessels and capillaries. Scale bar: 100 μm for αSMA and 50 μm for CD31. **b.** H&E stain and quantification (n=3; mean ± s.d.) of dynamic construct thickness before *in vivo* implantation (pre-impl) and after 2 and 15 days of implantation (2d and 15d post-impl). Scale bar: 100 μm. *: $p<0.05$; N: native diaphragm; DYN: dynamic construct.
