# Supplementary material for: Customized bioreactor enables the production of 3D diaphragmatic constructs influencing matrix remodeling and fibroblast overgrowth
Source: NPJ Regen Med. 2022 Apr 25;7:25. doi: 10.1038/s41536-022-00222-x (PMC9038738; doi:10.1038/s41536-022-00222-x)
Supplement: Supplementary file 3 — REPORTING SUMMARY [file 41536_2022_222_MOESM3_ESM.pdf]

nature portfolio

Corresponding author(s): Martina Piccoli
Piero Pavan

Last updated by author(s): Dec 20, 2021

Reporting Summary

Nature Portfolio wishes to improve the reproducibility of the work that we publish. This form provides structure for consistency and transparency in reporting. For further information on Nature Portfolio policies, see our Editorial Policies and the Editorial Policy Checklist.

Statistics

For all statistical analyses, confirm that the following items are present in the figure legend, table legend, main text, or Methods section.

n/a	Confirmed	
☐	☒	The exact sample size (n) for each experimental group/condition, given as a discrete number and unit of measurement
☐	☒	A statement on whether measurements were taken from distinct samples or whether the same sample was measured repeatedly
☐	☒	The statistical test(s) used AND whether they are one- or two-sided<br>*Only common tests should be described solely by name; describe more complex techniques in the Methods section.*
☒	☐	A description of all covariates tested
☐	☒	A description of any assumptions or corrections, such as tests of normality and adjustment for multiple comparisons
☒	☐	A full description of the statistical parameters including central tendency (e.g. means) or other basic estimates (e.g. regression coefficient) AND variation (e.g. standard deviation) or associated estimates of uncertainty (e.g. confidence intervals)
☒	☐	For null hypothesis testing, the test statistic (e.g. F, t, r) with confidence intervals, effect sizes, degrees of freedom and P value noted<br>*Give P values as exact values whenever suitable.*
☒	☐	For Bayesian analysis, information on the choice of priors and Markov chain Monte Carlo settings
☒	☐	For hierarchical and complex designs, identification of the appropriate level for tests and full reporting of outcomes
☒	☐	Estimates of effect sizes (e.g. Cohen's d, Pearson's r), indicating how they were calculated

Our web collection on statistics for biologists contains articles on many of the points above.

Software and code

Policy information about availability of computer code

Data collection	Microarray data analyses were performed with TAC Software v. 4.0.2.15 and normalized by R Bioconductor package (www.r-project.org) using Robust Multi-Array Average (RMA).<br>Immunofluorescence data were collected using LAS-AF software (Leica).
Data analysis	Statistical analyses were performed using GraphPad Prism 6v.<br>Gene Set Enrichment Analysis (GSEA) was performed using GSEAv2.0 and R version 4.0.2<br>Immunofluorescence analyses and goodness were performed using ImageJ software 1.52v.<br>FEM analyses were performed using ABAQUS CAE (version 2021, Dassault Systems).<br>Calcium transient profiles were quantified using Matlab 2021a.<br>Figure panels were made using Adobe Photoshop 22.2.0.

For manuscripts utilizing custom algorithms or software that are central to the research but not yet described in published literature, software must be made available to editors and reviewers. We strongly encourage code deposition in a community repository (e.g. GitHub). See the Nature Portfolio guidelines for submitting code & software for further information.

Data

Policy information about availability of data

All manuscripts must include a data availability statement. This statement should provide the following information, where applicable:

- Accession codes, unique identifiers, or web links for publicly available datasets
- A description of any restrictions on data availability
- For clinical datasets or third party data, please ensure that the statement adheres to our policy

Data described in the manuscript are available from the corresponding authors on reasonable request.

Field-specific reporting

Please select the one below that is the best fit for your research. If you are not sure, read the appropriate sections before making your selection.

☒ Life sciences ☐ Behavioural & social sciences ☐ Ecological, evolutionary & environmental sciences

For a reference copy of the document with all sections, see nature.com/documents/nr-reporting-summary-flat.pdf

Life sciences study design

All studies must disclose on these points even when the disclosure is negative.

Sample size	Sample size for every experiment performed and image acquired is presented in the figure legends.<br>For imaging data, images in the manuscript are representative images of a minimum n=3 experiments.<br>For quantitative analyses, no sample size calculation was performed but the sample size / replicate number was chosen in order to provide sufficient data points for the determination of measures of central tendency, variance, and parametric vs non-parametric distribution of the data.<br>Statistical significance of reported results was assessed by statistical tests during data analyses, as indicated in Methods section.
Data exclusions	No data were excluded from the analyses.
Replication	Cell and tissue-like culture experiments were replicated within our laboratory by at least two independent operators (E.M. and E.C.).
Randomization	When tissue-like cultures were prepared in replicate, the samples intended for static and dynamic culture were randomly assigned.<br>Mice that recieved dynamic constructs or acellular dECM were randomly assigned.
Blinding	The investigator analyzing the experiment was not the same investigator performing the experiment for all immunofluorescence and qPCR data. All immunofluorescence quantification were performed in blind by two investigators.

Reporting for specific materials, systems and methods

We require information from authors about some types of materials, experimental systems and methods used in many studies. Here, indicate whether each material, system or method listed is relevant to your study. If you are not sure if a list item applies to your research, read the appropriate section before selecting a response.

Materials & experimental systems

n/a	Involved in the study	
☐	☒	Antibodies
☐	☒	Eukaryotic cell lines
☒	☐	Palaeontology and archaeology
☐	☒	Animals and other organisms
☒	☐	Human research participants
☒	☐	Clinical data
☒	☐	Dual use research of concern

Methods

n/a	Involved in the study	
☒	☐	ChIP-seq
☐	☒	Flow cytometry
☒	☐	MRI-based neuroimaging

Antibodies

Antibodies used	aSMA (Mouse; ab-7817) 1:100 Abcam; Muscle Actin (Mouse; AF488) 1:100 Invitrogen; Alfa Sarcomeric Actinin 2 (Mouse; A7811) 1:100 Sigma-Aldrich; Fibronectin (Rabbit; ab2413) 1:100 Abcam; Human Nuclei (Mouse; MAB1281) 1:200 Millipore; KI67 (Rabbit; ab-15580) 1:100 Abcam; Laminin (Rabbit; L9393) 1:200 Sigma-Aldrich; Laminin α2 (Rat; L0663) 1:100 Sigma-Aldrich; Laminin α5 (Mouse; MA1-83286) 1:100 Termo Fisher; MHC (Mouse; MAB4470) 1:100 R&D Systems; MYOD (Mouse; M3512) 1:50 Dako; Myogenin AF488 (Mouse; 53-5643-82) 1:80 Invitrogen; TE7 (Mouse; CBL271) 1:100 Millipore; Type 1 Collagen (Rabbit; ab34710) 1:100 Abcam; Type 4 Collagen (Rabbit; ab6586) 1:100 Abcam; Human-specific Type 1 Collagen (Rabbit; HPA011795) 1:100 Sigma-

Merk; Human-specific Type 4 Collagen AF647 (Mouse; 51-9871-82) 1:100 Invitrogen.
Anti-Rabbit 488 1:200 Life Technologies; Anti-Rat 488 1:200 Life Technologies; Anti-Rabbit 594 1:200 Life Technologies; Anti-Mouse 594 1:200 Life Technologies; Anti-Rat 568 1:200 Life Technologies.
The same list is included in supplementary Information.

Validation

Antibodies were validated in primary human skeletal muscle cells and mouse diaphragms, and using information on the manufacturer's website.

Eukaryotic cell lines

Policy information about cell lines

Cell line source(s)	Primary normal human skeletal myoblasts (hSkMC) were purchased from Gibco-Fischer Scientific.<br>Primary dermal fibroblast (hFb, PCS-201-012) were purchased from ATCC.
Authentication	All the cell lines are authenticated by the producing company and commercially available.
Mycoplasma contamination	All cell lines used in this work were tested monthly and always tested negative for Mycoplasma.
Commonly misidentified lines<br>(See ICLAC register)	Not relevant.

Animals and other organisms

Policy information about studies involving animals; ARRIVE guidelines recommended for reporting animal research

Laboratory animals	Diaphragm muscles were obtained from 3 months old C57BL/6j and Rag2-/-gc-/- mice.
Wild animals	This study did not involve wild animals.
Field-collected samples	Animals were housed following these parameters:<br>A 12 light/12 dark cycle was used;<br>Temperatures of ~18-23°C with 40-60% humidity;<br>Water was accessible at all times;<br>Fat content ranges from 4% to 11%;<br>Mice handling was gently and as little as possible, especially when females were pregnant, close to delivering, or have new litters.
Ethics oversight	All in vivo experiments (as described in Materials and Methods section) were performed using Protocols N. 1103/2016 and N. 418/2020-PR approved by Animal wellness local ethics committee (Organismo per il Benessere Animale - OPBA, University of Padova and Fondazione Istituto di Ricerca Pediatrica Città della Speranza) and Italian Ministry of Health.

Note that full information on the approval of the study protocol must also be provided in the manuscript.

Flow Cytometry

Plots

Confirm that:

☒ The axis labels state the marker and fluorochrome used (e.g. CD4-FITC).

☒ The axis scales are clearly visible. Include numbers along axes only for bottom left plot of group (a 'group' is an analysis of identical markers).

☒ All plots are contour plots with outliers or pseudocolor plots.

☒ A numerical value for number of cells or percentage (with statistics) is provided.

Methodology

Sample preparation	Cells were detached from the culture dish, washed with medium, incubated with anti-CD56 and 7 amino-actinomycin D (7AAD), washed two times and then analysed.
Instrument	Cells were analysed using Accury C6 (Beckton Dickinson) flow cytometer.
Software	Data were analysed with Accury C6 software.
Cell population abundance	Cells were not sorted, but only analysed for the expression of CD56 marker.
Gating strategy	The first gate was drawn on FSC/SSC plot excluding debris and too much little events. The second gate was drawn on 7AAD negative cells (named live cells), and the CD56 expression was evaluated on this gate for all the analysed samples.

☐ Tick this box to confirm that a figure exemplifying the gating strategy is provided in the Supplementary Information.
